# Recurrent Glioblastomas Reveal Molecular Subtypes Associated with Mechanistic Implications of Drug-Resistance

**DOI:** 10.1371/journal.pone.0140528

**Published:** 2015-10-14

**Authors:** So Mee Kwon, Shin-Hyuk Kang, Chul-Kee Park, Shin Jung, Eun Sung Park, Ju-Seog Lee, Se-Hyuk Kim, Hyun Goo Woo

**Affiliations:** 1 Department of Physiology, Ajou University School of Medicine, Suwon, Republic of Korea; 2 Graduate School of Biomedical Science, Ajou University School of Medicine, Suwon, Republic of Korea; 3 Department of Neurosurgery, Korea University College of Medicine, Seoul, Republic of Korea; 4 Department of Neurosurgery, Seoul National University College of Medicine, Seoul, Republic of Korea; 5 Department of Neurosurgery, Chonnam National University Research Institute of Medical Sciences, Chonnam National University Hwasun Hospital & Medical School, Gwangju, Republic of Korea; 6 Institute for Medical Convergence, Yonsei University College of Medicine, Seoul, Korea; 7 Department of Systems Biology, The University of Texas M. D. Anderson Center, Houston, Texas, United States of America; 8 Department of Neurosurgery, Ajou University School of Medicine, Suwon, Republic of Korea; University of Navarra, SPAIN

## Abstract

Previously, transcriptomic profiling studies have shown distinct molecular subtypes of glioblastomas. It has also been suggested that the recurrence of glioblastomas could be achieved by transcriptomic reprograming of tumors, however, their characteristics are not yet fully understood. Here, to gain the mechanistic insights on the molecular phenotypes of recurrent glioblastomas, gene expression profiling was performed on the 43 cases of glioblastomas including 15 paired primary and recurrent cases. Unsupervised clustering analyses revealed two subtypes of G1 and G2, which were characterized by proliferation and neuron-like gene expression traits, respectively. While the primary tumors were classified as G1 subtype, the recurrent glioblastomas showed two distinct expression types. Compared to paired primary tumors, the recurrent tumors in G1 subtype did not show expression alteration. By contrast, the recurrent tumors in G2 subtype showed expression changes from proliferation type to neuron-like one. We also observed the expression of stemness-related genes in G1 recurrent tumors and the altered expression of DNA-repair genes (i.e., *AURK*, *HOX*, *MGMT*, and *MSH6*) in the G2 recurrent tumors, which might be responsible for the acquisition of drug resistance mechanism during tumor recurrence in a subtype-specific manner. We suggest that recurrent glioblastomas may choose two different strategies for transcriptomic reprograming to escape the chemotherapeutic treatment during tumor recurrence. Our results might be helpful to determine personalized therapeutic strategy against heterogeneous glioma recurrence.

## Introduction

Glioblastoma is the most aggressive and frequent primary brain tumor with dismal prognosis [1, 2]. The incurable outcome of the glioblastoma is largely due to high recurrence rate even after total resection of glioblastoma mass [2, 3]. Also, highly infiltrative characteristics of the glioblastoma make it impossible to dissect tumor tissues completely and the majority of glioblastomas are destined to recur less than 6 months after surgical resection [4, 5]. Therefore, new diagnostic and therapeutic strategies for tumor recurrence might be required to improve clinical outcomes of patients.

Previously, numerous genomic profiling studies have addressed the marked heterogeneity of glioblastomas [6–9]. Particularly, The Cancer Genome Atlas (TCGA) project recognized four distinct molecular subtypes of proneural, neural, classical, and mesenchymal, which are different in response to aggressive therapies [10, 11]. In addition, an earlier study has shown that about one third (8 out of 26) of the recurrent glioblastomas shifted their subtypes toward mesenchymal subtype [12]. However, there is a conflicting observation that the molecular subtypes are not altered by recurrence [11], remaining the mechanisms for tumor recurrence still unveiled. With this concern, in the present study, we re-evaluated the alteration of the molecular phenotypes of recurrent glioblastomas by comparing gene expression profiles of paired primary and recurrent glioblastomas. We could identify two different modes of transcriptomic reprograming during recurrence of glioblastomas, and which implied subtype-specific mechanisms for the acquisition of drug-resistance by tumor recurrence. Our analysis may provide new mechanistic and clinical insights on the recurrent glioblastoma management.

## Materials and Methods

### Preparation of glioblastoma specimens

The frozen tissues of 43 glioblastomas from 28 patients who had received surgery were obtained from four different hospitals of Ajou University Hospital, Korea University Hospital, Seoul National University Hospital, and Chonnam National University Hospital. The study was approved by the Institutional Review Board of Ajou University Hospital, Institutional Review Board of Korea University Hospital, Institutional Review Board of Chonnam University Hospital, and Institutional Review Board of Seoul National University Hospital, and obtained written informed consents from donors. All patients underwent surgical resection, the degree of which was categorized as <50%, 50 to 90%, or gross total resection (no distinct residual tumor) based on comparison of pre- and postoperative magnetic resonance (MR) images obtained <48 hours after surgery. All the primary tumors had been confirmed with pathologic examination following surgical resection and treated with the same protocol including concurrent radio-chemotherapy with treatment of temozolomide (TMZ) for more than three cycles [2]. A total of 25 primary tumors and 18 recurred tumors were used for gene expression profiling. To compare the primary and recurrent tumors, 15 pairs of primary and recurred glioblastomas from the same patients were included in this study. Tumor volume was calculated with 4/3pi(a x b x c) cm^3^, (a, b and c is radius in each direction) and the degree of necrosis was examined with hypointense region of T1 signal surrounded by a contrast enhanced region representing viable tumor in magnetic resonance images. Necrosis was graded according to the following previously described system: grade 0, no necrosis apparent on the magnetic resonance imaging scan; grade I, amount of necrosis is <25% of the tumor volume; grade II, amount of necrosis is between 25% and 50% of the tumor volume; grade III, amount of necrosis is >50% of the tumor [13]. In addition, ependymal involvement was defined as contrast enhancement of periventricular region in T1 images.

### Gene Expression Profiling

Total RNA was extracted from frozen tumor section (10 to 15 mg: mirVana^TM^ miRNA isolation Kit, Ambion, AM1560) based on the manufacturer’s guideline. The quantification of RNA was performed using the Nanodrop ND-1000 spectrophotometer (Thermo-Fisher) and the quality of total RNA was evaluated using the RNA 6000 nano kit (Agilent Technologies, 5067–1513) and the Agilent 2100 Bioanalyzer (Agilent Technologies). Cut off value of the integrity of RNAs used in RNA amplification is over 7.0 in the RIN level. For microarray experiments, five hundred (500) ng of total RNA per sample was used for complement RNA (cRNA) production by the Illumina TotalPrep RNA amplification kit (Ambion, IL1791) according to the provided protocol. A total of 750 ng cRNA was used for hybridization to a human HT12-v4 Illumina Beadchip gene expression array (Illumina) according to the manufacturer’s protocol. The arrays were scanned and fluorescence signals obtained using Illumina bead Array Reader confocal scanner, and obtained the intensity data with GenomeStudio software. Raw data were normalized by applying log 2 transformation, quantile normalization, and gene and array centering. All of the data processing was performed using the R/Bioconductor packages.

For validation analysis, two independent gene expression data of REMBRANDT [14] and TCGA [11] were obtained from their websites, respectively. To integrate different data set, preprocessing of each data set was applied including log 2 transformation, quantile normalization, and gene and array centering.

### Classification of subtypes

For subtype prediction, three independent methods of unsupervised hierarchical clustering, consensus clustering [15], and nearest template prediction (NTP) [16] were applied. For consensus clustering, hierarchical clustering with the distance metric by Pearson (1—Pearson correlation) was used. For K ranging from 2 to 6, hierarchical clustering was run over 10,000 iterations with a sub-sampling ratio of 0.8 for estimating the consensus matrix. For the purpose of visualization and cluster identification, hierarchical clustering with the Pearson (1—Pearson correlate) distance metric and the average linkage option was applied to the estimated consensus matrix. NTP analysis was performed using GenePattern software (http://www.genepattern.org). The classifiers for the four class subtypes in TCGA dataset [11] were applied and annotated with the numeric code representing the unique subtype that each gene represents (1, 2, 3, 4, 5 for proneural, neural, classical, mesenchymal, and unclassified subtypes) with statistical significance of Bonferroni p value < 0.05 with 1,000 resampling bootstrap test.

### Gene set analysis

The functional profiling of biological function and signaling pathways were performed using DAVID software [3]. Coordinated gene regulation was identified using gene set enrichment analysis (GSEA: http://www.broadinstitute.org/gsea) with nominal P-value cutoff of 0.0001.

### Data deposition

The raw data of the microarray experiments are available in the GEO database (http://www.ncbi.nlm.nih.gov/geo) with accession number **GSE62153**.

## Result

### Gene expression profiling reveals two subtypes of recurrent glioblastoma

A total of 28 glioblastoma patients were enrolled for this study. The patients were treated with temozolomide (TMZ) after surgical resection. Overall, the progression free survival time (PFS) of the patients was ranged from 5 to 62.4 months, and the median PFS and median overall survival time were 10.75 and 20.50 months, respectively. Detailed clinical information of the patients were summarized in Table 1.

**Table 1:** Clinical features in primary and recurrent glioblastoma patients.

Features	Total patients (n = 28)
Age (years)	51.18 ± 14.20
Median	50.5 (24~77)
<50	14/28 (50%)
51–60	5/28 (18%)
61–70	6/28 (21%)
>70	3/28 (11%)
Gender	
Male: Female	14:14
Hospital management	
AJOU/KU/CNU/SNU	9/6/8/5
Tumor Volume (cm^3^)	45.65 ± 21.35
Median	46.20 (19.6 ~116.4)
MR necrosis (%)	
No necrosis	2/28 (7%)
<25%	8/28 (29%)
25–50%	4/28 (14%)
>50%	14/28 (50%)
Ependymal involvement	
YES	10/28 (36%)
NO	18/28 (64%)
Initial KPS	
90–100	15/28 (53%)
70–80	11/28 (40%)
<70	2/28 (7%)
Included glioblastoma tissue in this study	
Primary glioblastoma only	10/28 (32%)
Recurred glioblastoma only	3/28 (11%)
Both primary and recurred glioblastoma	15/28 (54%)
Initial resection	
Gross total resection	17/28 (60%)
Subtotal resection (50 ~90%)	8/28 (29%)
Partial resection (<50%)	3/28 (11%)
Progression free survival (months)	14.91 ± 12.17
Median	10.75 (5~62.4)
Overall Survival (months)	26.16 ± 18.20
Median	20 (9~ 78)

AJOU: Ajou University Hospital, KU: Korea University Hospital, CNU: Chonnam National University Hospital, SNU: Seoul National University Hospital, MR: magnetic resonance; KPS: Karnofsky Performance Status Scale.

**Fig 1:**
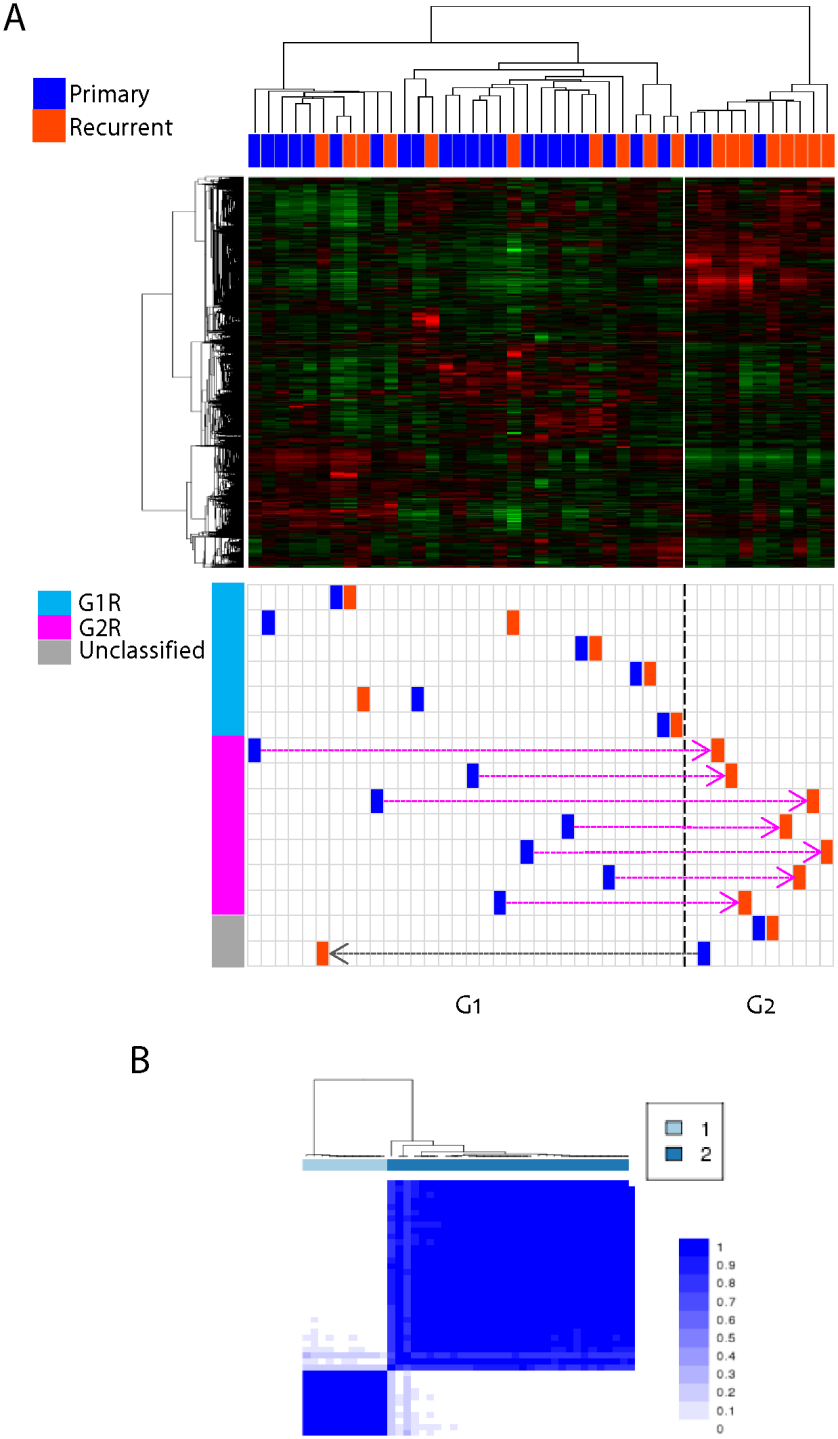
Gene expression profiling of primary and recurrent glioblastomas. (**A**) Unsupervised clustering analysis showed two distinct clusters of G1 and G2 tumors (*top*). The primary and recurrent glioblastoma were marked with dark blue and dark orange color, respectively (*bottom*). The 15 paired primary and recurrent glioblastomas were marked based on the defined two clusters, G1 and G2. (**B**) Heatmap shows the consistency of the consensus clustering analysis with k = 2.

To characterize the gene expression patterns of the primary and recurrent glioblastomas, we performed gene expression profiling of the 43 tumor tissues which included the 15 cases of paired primary and recurrent glioblastomas and 13 unpaired tumor tissues. First, to demonstrate the overall gene expression patterns, unsupervised clustering analysis was performed using most variable 4,650 genes with standard deviation (S.D.) greater than 0.5. This revealed two distinct clusters of G1 (n = 32) and G2 (n = 11) subtypes (Fig 1A, *top*). The consistency of the cluster was validated by applying consensus clustering algorithm implemented in Genepattern software, which could confirm the robustness of the two clusters showing the same two clusters (Fig 1B). When we examined the distribution of primary and recurrent glioblastomas from the cluster result, most of the primary glioblastomas were classified into the G1 cluster. However, the recurrent glioblastomas were found in both G1 (n = 10) and G2 (n = 8) clusters. Recurrent glioblastomas were more frequent in G2 cluster with statistical significance (P = 0.031, odd ratio = 5.60, Fisher’s exact test), implying the enriched expression of recurrence-related genes in the G2 tumors.

**Fig 2:**
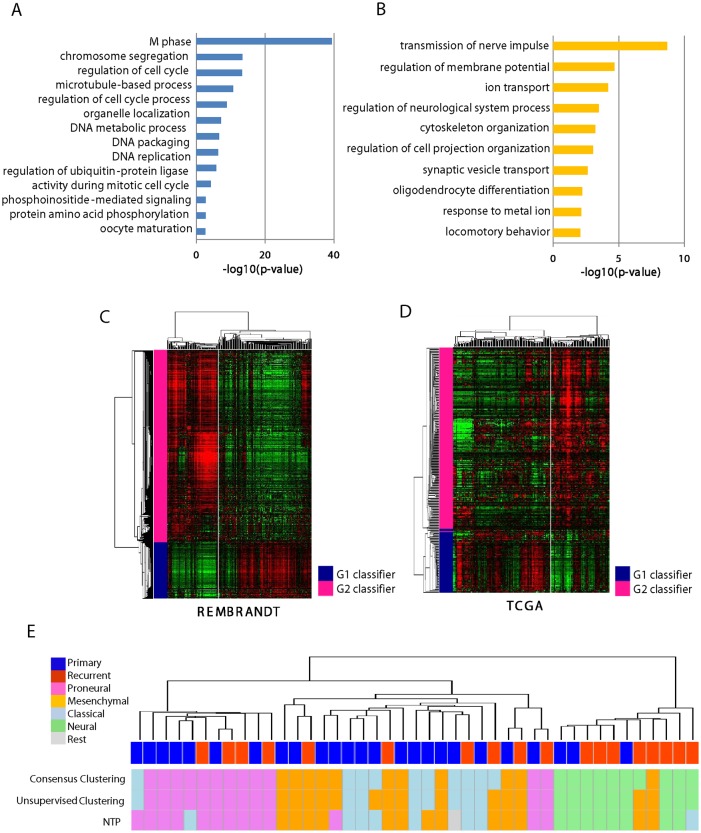
Functional characteristics of G1 and G1 subtypes. (**A-B**)The enriched GO terms of the subtype classifiers are indicated. The significance of the enrichment is plotted as value of—log_10_(p-value). (**C-D**) Unsupervised hierarchical clustering analysis showed the conserved expression patterns of the classifiers in independent data set, REMBRANDT (**C**) and TCGA (**D**). (**E**) Gene expression similarity with the four subtypes of TCGA are evaluated by applying three different methods of consensus clustering, unsupervised clustering, and nearest template prediction (NTP) as described in the **Materials and Methods**. The primary and recurrent tumors are indicated with different colors. The predicted four classes of proneuronal, mesenchymal, classical, neural type are indicated. Unclassified tumors are indicated as rest.

To address the functional characteristics of the clusters, we identified differentially expressed genes between G1 and G2 tumors as subtype classifiers (*i*.*e*., 94 up-regulated and 318 down-regulated genes, respectively) by applying permutation t-test (*P < 0*.*001*) and fold differences greater than two (S1 Table). The genes expressed in the G1 cluster were significantly enriched with cell cycle-related gene functions such as M phase, chromosome segregation, cell cycle regulation, and DNA metabolic process, while the genes expressed in the G2 cluster were enriched with neuron development-related genes such as neuron projection morphogenesis, regulation of cell projection organization, ion homeostasis (Fig 2). Comparing to the previous TCGA subtypes [10, 11], this result suggests that the G1 tumors are similar to proliferation type and the G2 tumors are similar to neuronal type, respectively. The expression of neuronal differentiation-related genes might be a key feature of the transcriptomic switch from primary G1 tumors to the paired recurrent G2 tumors.

Next, we compared the gene expression changes between the 15 paired primary and recurrent glioblastomas. Remarkably, we found two distinct behaviors of gene expressions in the recurrent glioblastomas compared to those in the paired primary tumors (Fig 1A, *bottom*). A total of 7 out of 15 recurrent glioblastomas showed the cluster migration from G1 to G2 subtype. The other 6 recurrent tumors resided in the same cluster with the paired primary tumors. Exceptionally, only one case of recurrent tumor showed opposite migration from G2 to G1 cluster, and one case of G2 recurrent tumor did not migrate to other cluster. These results suggest that the recurrent glioblastomas might have at least two distinct patterns of molecular changes after being recurred. The G1 type recurrent tumors (G1R, n = 6) showed no subtype migration, while the G2 type recurrent tumors (G2R, n = 7) showed subtype migration from G1 to G2 subtype (see S2 Table).

### Validation of the subtype classifiers of glioblastoma using independent data sets

As shown above, the G1 and G2 classification is clearly associated with the expression migration during tumor recurrence. To further validate the robustness and the significance of our classification, we examined the expression pattern of our subtype classifiers in the independent two data sets of REMBRANT [14] and TCGA [10]. We could observe that the expressions of the subtype classifiers were well conserved in both data sets stratifying G1-like and G2-like subtypes, respectively (Fig 2C and 2D). This result indicated that our subtype classifiers were well conserved independent of patient cohorts and/or data platforms, and might be useful in predicting the subtypes of tumor recurrence. However, when we evaluated the clinical outcomes of the G1-like and G2-like subtypes by Kaplan-Meir plot analysis, there was no significant difference of overall survival between the groups (S1 Fig). This may indicate that our classification does not represent a prognostic sub-classification, but a classification for different mode of mechanistic pathways for tumor recurrence.

Confirming the conserved expression of the classifiers in the independent datasets, we next evaluated the relationship between our subtypes and the TCGA subtypes of mesenchymal, proneural, classical, and neural type [11]. Prediction of the subtypes was performed on the integrated data set of TCGA and ours using the overlapped genes with variable expressions (n = 4,378, S.D. > 0.5). By applying three different classification methods of unsupervised hierarchical clustering, consensus clustering, and nearest template prediction (NTP) on the integrated data set (for details see the Materials and Methods), we could successfully re-identify the four subtypes, respectively (S2 Fig and S3 Table). Unsupervised clustering analysis with the integrated data set could reveal four classes which were compatible with the previous TCGA subtypes (S2A Fig). Consensus clustering analysis also showed four distinct expression subtypes (S2B and S2C Fig). When we compared these classification results with our subtypes of G1 and G2, we could observe that the G2 tumors had similar expression pattern to that of neural subtype, while the G1 tumor was similar to those of other three groups of mesenchymal, proneuronal, and classical subtypes (Fig 2E). This result was consistent with the result of GO analysis (see Fig 2B). Taken together, we could suggest that the recurrent glioblastomas have at least two different patterns of G1 and G2 subtype. The G2 subtype is similar to neural subtype, while the G1 subtype is likely to be mixed with the other types.

### Expression of stemness and drug-resistance-related genes might be involved in the subtypes of recurrence glioblastomas

**Fig 3:**
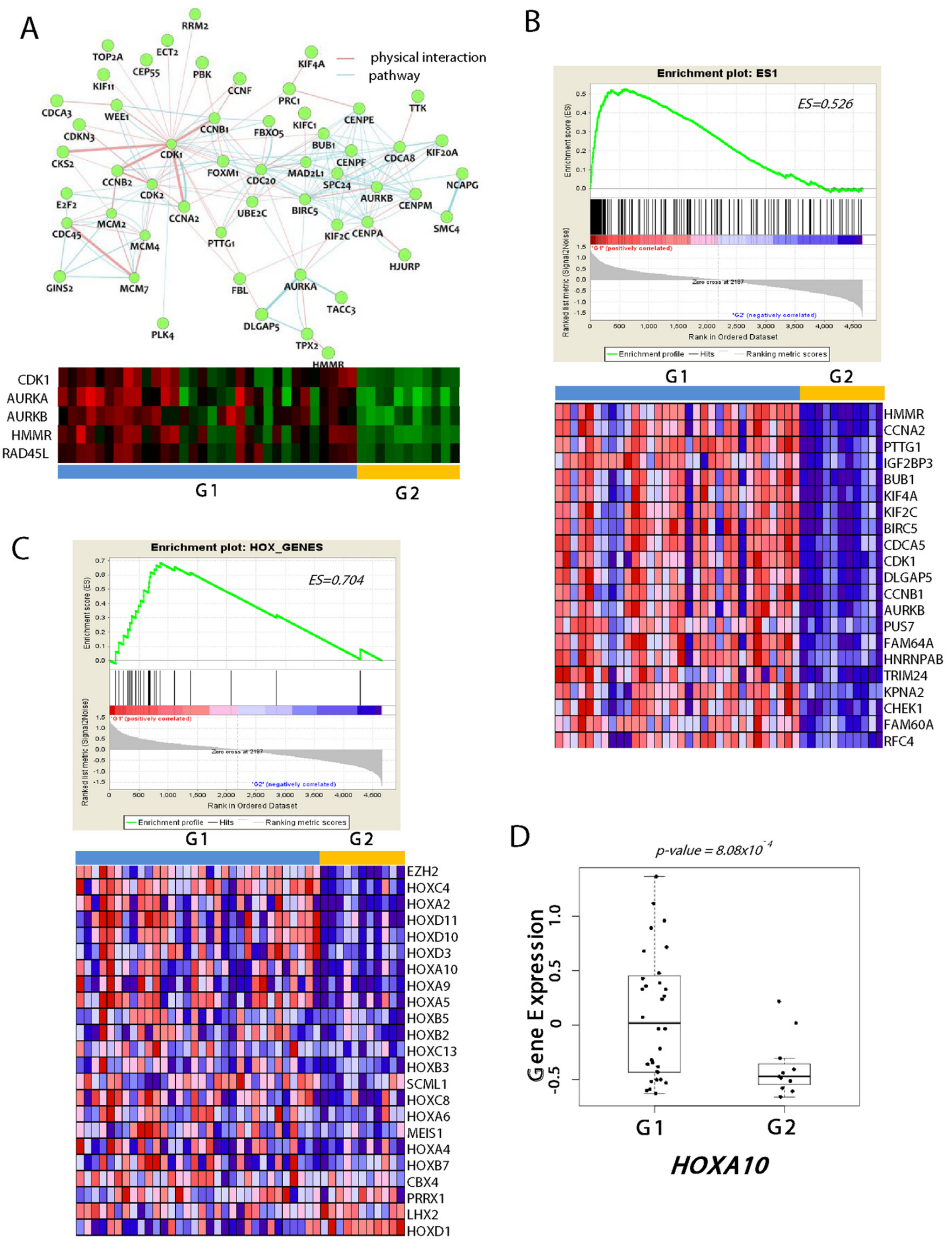
Expression of stemness-like traits in G1 recurrent tumors. (**A**) Network analysis using G1 signature genes reveals the CDK and AURK as the key hub genes (*top*). Pathway (*light blue*) and physical interactions (*light pink*) are indicated with different colors. The heatmap of the expression of the key hub genes (*CDK1*, *AURKA*, *AURKB*, *HMMR*, *RAD45L*) are plotted (*bottom*) (**B**) The GSEA result show the enrichment of the ES1 signature (*top*) and the expression of the top 20 differentially expressed genes are shown (*bottom*) (**C**) The plots showed the enrichment scores (ES) for the HOX_GENE signature (*top*) and their expression heatmap is shown (*bottom)*. (**D**) The expression of HOX10a in G1 and G2 tumors are plotted. Statistical significance is calculated using Welch Two Sample T-test.

To further gain an insight on the differential molecular determinants in the G1 and G2 clusters, a network analysis was applied by using GeneMANIA software (version 3.2)[17]. This revealed *CDK1* (cyclin-dependent kinase 1), *AURKA* (aurora kinase A), and *AURKB* (aurora kinase B) as key hub regulators for G1 tumors (Fig 3A). Indeed, *AURKA* is well known to play an important function in tumor development, progression, and patient survival [18–21]. Moreover, *AURKA* is strongly correlated with survival of glioma stem cells [22]. *AURKB* has also been associated with TMZ susceptibility [23] and aggressive outcomes of glioblastomas [24]. *CDK1* is also known to play regulatory roles in the self-renewal of mouse embryonic stem cells [25] as well as for cell survival of glioblastoma [26]. These findings may support that the selective targeting of these genes for G1 recurrent tumors might be beneficial in the clinic.

In addition, when we performed gene set enrichment analysis, the G1 tumors showed significant enrichment of stemness-related genes, ES1 (ES = 0.526, P-value < 0.001, False Discovery Rate (FDR) < 0.001) which has been identified previously elsewhere [27]. Among the ES1 genes, *HMMR* (Hyaluronan-mediated motility receptor) was top ranked (Fig 3B), suggesting its pivotal role in the stem cell-like characteristics of G1 tumors. *HMMR* has recently been reported to express in the gliomas and to play a crucial role in self-renewal and tumorigenic potential of glioblastoma stem cells [28]. Supporting this, we also observed that *HOX* genes were enriched and differentially expressed (ES = 0.704, P-value < 0.001, FDR < 0.001) in the G1 tumors (Fig 3C), which have been notified as “self- renewal”-associated genes in gliomas [29, 30]. Of these, *HOXA10* showed marked over-expression in G1 tumors (Fig 3D). *HOXA10* has been known to involve in homologous recombinant DNA repair pathway [31], playing a key role in TMZ resistance in glioblastomas [29]. Congruent with these findings, the G1 tumors showed significant enrichment of the DNA_REPAIR genes (ES = 0.686, P-value < 0.001, FDR < 0.001, S3A Fig). Therefore, we could suggest that resistance to the chemotherapeutic agent may be attributed by the inherited stem-cell-like characteristics of the G1 tumors. The self-renewal properties and the activated DNA repair system (*e*.*g*., *HOXA10*) might be responsible for the relapse of the recurrent G1 glioblastomas after resection and adjuvant treatment.

### Differential expression of *MGMT* and *MSH6* genes in the subtypes of recurrent glioblastomas

**Fig 4:**
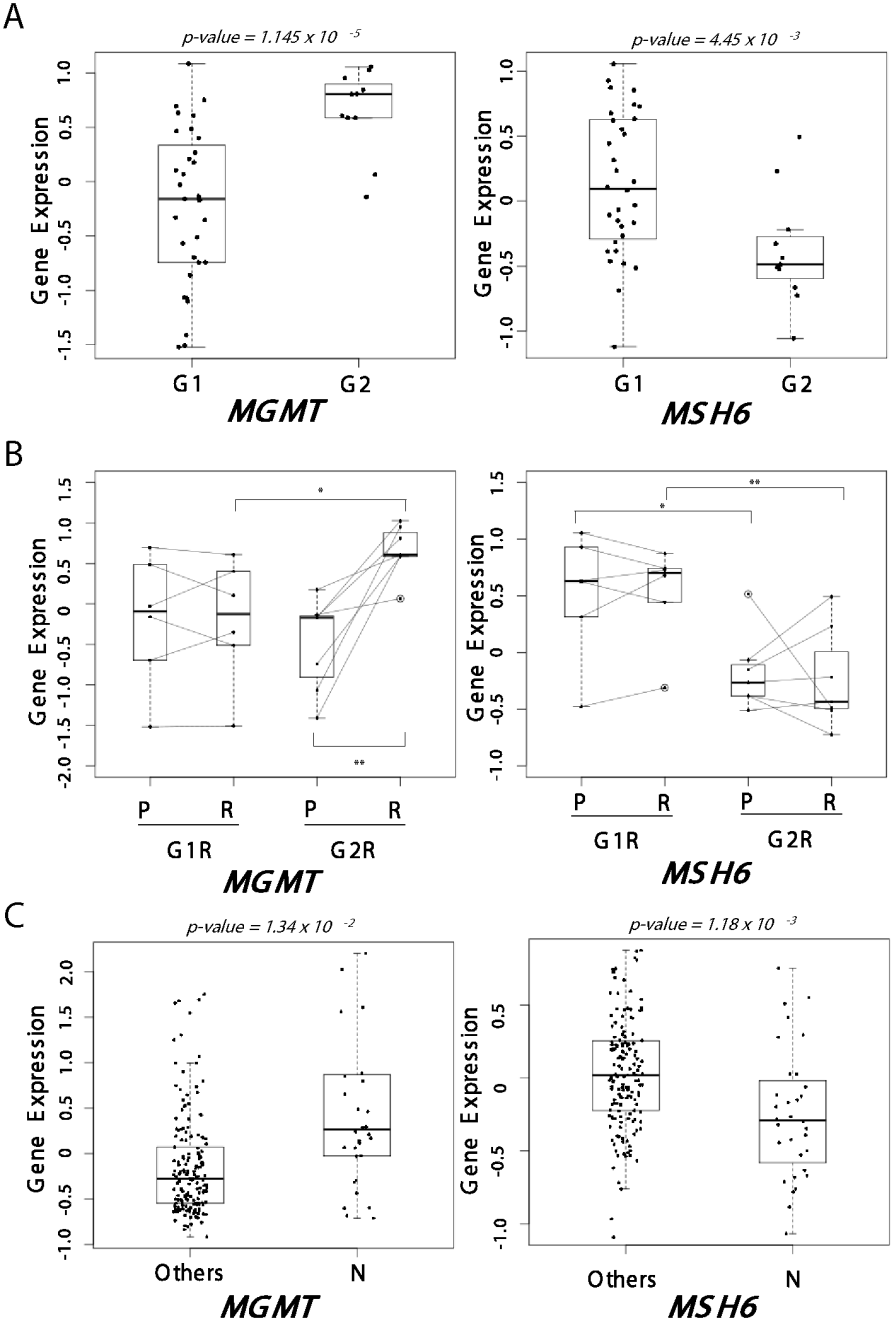
Differential expression of MGMT and MSH6 genes between G1 and G2 tumors. (**A**) The expressions of *MGMT* (*left*) and *MSH6* (*right*) were evaluated in G1 and G2 tumors. **(B)** Paired comparison of *MGMT* (*left*) and *MSH6* (*right*) expressions between primary (P) and paired recurrent (R) tumors. Traced lines indicate the expression changes between primary and paired recurrent tumors. (**C**) The comparison of *MGM*T (*left*) and *MSH6* (*right*) expressions between the neural subtype (N) and the other subtypes. The statistical significance is evaluated using Welch Two Sample t-test (*significant at *P < 0*.*05*, **significant at *P< 0*.*005*).

As the glioblastoma subtypes were associated with drug-resistance, we hypothesized that different tactics to escape the chemotherapeutics might be involved in recurrent glioblastomas of each subtype. TMZ has been currently emerged as a new standard regimen in glioblastoma. Previous studies have demonstrated that the therapeutic effects of TMZ might be restricted to the patients whose *MGMT* (O-6-methylguanine–DNA methyltransferase) promoters were methylated [32, 33], which might be due to the *MGMT* repairing DNA-alkylated adducts could diminish the TMZ cytotoxicity induced by O6-methylguanine-DNA adducts [34]. In addition, it has been suggested that MGMT-independent DNA repair pathway could affect TMZ effectiveness [35–37]. Indeed, it has been demonstrated that the activation of DNA mismatch repair (MMR) system could promote TMZ resistance [35–38]. With respect to this, we examined the expression of both *MGMT* and MMR genes (*i*.*e*., *MLH1*, *MSH2*, and *MSH6*). *MGMT* was significantly up-regulated in the G2 subtype than the G1 subtype (*P = 1*.*145 x 10* ^*−5*^, Fig 4A). By contrast, the *MSH6* expression was significantly down-regulated in G2 subtype implying their decreased activity of MMR pathway (*P = 4*.*45 x 10* ^*−3*^). When we compared the paired primary and recurrent tumors, marked change of *MGMT* expression could be observed in recurrent G2 (G2R) but not in recurrent G1 (G1R) tumors (*P<0*.*005*, Fig 4B, *left* **)**. Vice versa, *MSH6* showed significant lower expression in the G2R tumors compared to the G1R tumors (*P = 0*.*0098*). Taken together, our results strongly suggest that the G2 but not G1 tumors may acquire TMZ tolerance via altered expression of *MGMT* and MMR pathway genes.

As the G2 subtype showed similar expression pattern with neural subtype (see Fig 2), we next compared the expression of *MGMT* and *MSH6* among the subtypes of TCGA data. As expected, the neural subtype showed significant overexpression of *MGMT* (*P = 1*.*18 x 10* ^*−3*^, Fig 4C, *left*) and down-expression of *MSH6* (*P = 1*.*34 x 10* ^*−2*^, Fig 4C, *left*) compared to the other subtypes, respectively. When we compared the four subtypes of TCGA, the neural subtype showed the highest expression of *MGMT* and the lowest expression of *MSH6* compared to other subtypes (S4A and S4B Fig). These result may support our result showing the subtype-specific mechanism of TMZ resistance.

In addition, we further evaluated several possible mechanisms which have been addressed previously. Epigenetic silencing by promoter methylation of MGMT has been noticed to associate with survival benefit for TMZ treatment [33], however, we could not find any significant difference of the methylation of *MGMT* among the TCGA subtypes (S4C Fig). We also evaluated the expression of ABC family genes (*i*.*e*., *ABCC1*, *ABCC3*, *and ABCC5*) which have been known to play important roles in drug-resistance [39], but was not associated with the recurrence subtypes (S5 Fig). Furthermore, we examined the mutation status of several drug resistance-related genes (*i*.*e*.,*TP53*, *MGMT*, *MSH6*, *ABCC1*, *ABCC5*) in the recurrence subtypes predicted in TCGA (*i*.*e*., G1-like and G2-like tumors), but no significant difference was found (S4 Table). This might be due to relatively low frequency of mutations, suggesting that further large scale studies might be required to elucidate the mutation effects.

## Discussion

In this study, by performing integrative gene expression profile analyses, we have demonstrated that there are two distinct subtypes of transcriptomic reprograming during recurrence of glioblastomas. From the results, we could suggest that the distinct two different mechanisms might be involved in for the TMZ resistance in each subtype. The G1 recurrent tumors had similar expression with the paired primary tumors, which express stemness and DNA-repair related genes. By contrast, the G2 recurrent tumors showed gene expression migration acquiring neuron-like traits. This may reflect the two different mechanisms might be involved in the acquisition of the recurrence phenotypes. Further interrogation has revealed the differential expression of *MGMT* and *MSH6* between the subtypes (Fig 4B), which suggested the involvement of distinct mechanisms for TMZ resistance during recurrence of glioblastomas. The G1 tumors expressed the stem cell- related “self-renewal” signature including *HOX*_genes, stemness genes (ES1), *CDK*, and *AURKA/B* genes in both the paired primary and recurrent tumors. The G1 recurrent tumors didn’t show subtype migration by recurrence, indicating that the initial gene expression profiles were remained without change even after treatment and disease progression. Thus, the expression of stemness genes might be a possible explanation for the TMZ resistance in G1 recurrent tumors. On the other hand, the G2 tumors showed significant differential expression of *MGMT* and *MSH6* genes compared to the primary tumors. As an underlying mechanism for the TMZ resistance, it has been addressed that MGMT protein removes the methyl or chloroethyl damage at the O6 position of guanine [40]. In addition, the mismatch repair system (MMR) is also considered to be involved in the TMZ resistance, amending the DNA damage and base mismatches [41]. MMR recognizes unrepaired O6-methylated guanine adduct and induces cytotoxicity. Thus, inactivation of MMR may induce TMZ tolerance [34, 38]. In this regards, the G2 tumors showed the acquired expressions of *MGMT* and inactivation of MMR system genes (*MSH6*), which might be responsible for the acquisition of TMZ resistance.

It is interesting to find that the G2 recurrent tumors acquire neuron-like features. Indeed, we have previously demonstrated the xenografted tumors in the brain acquire neuron-like expression traits, mimicking neurogenesis during development [42]. This results showed the connection of tumors with brain microenvironment such as neighbor astrocytes can give rise to chemo-resistant nature of brain metastatic tumors. Congruently, our data strongly support that brain environment may contribute to the neuron-like transcriptional reprograming in G2 recurrent tumors.

In addition, we have shown in the previous study the high concordance between promoter methylation and gene expression profiles, suggesting the contribution of epigenetic events to transcriptome reprogramming [42]. This raises a possibility that the acquisition of neuron-like trait in the G2 subtype might be related with the methylation reprograming. However, we could not observe from TCGA data the associations between methylation status and the tumor recurrence subtypes. To address the roles of epigenetic reprogramming to the transcriptomic reprogramming during glioma recurrence accurately, further large scale studies with detailed methylation profiling might be needed.

Our study demonstrated the subtype-specific transcriptomic reprograming might occur during recurrence of glioblastomas. Also, our data imply that the transcriptome changes rather than transcriptome *per se* can be of great importance in the acquisition of tumor recurrence and TMZ resistance. Thus, we suggest that our discovery of the classifiers might be beneficial in predicting the therapeutic targets for transcriptomic reprograming during tumor recurrence. In parallel to transcriptomic alteration, recent studies have identified genomic landmarks of recurrent glioblastomas, including the increased TMZ-induced mutagenesis and the mutations in RB and Akt-mTOR pathways [43]. It has also been suggested that the distant appearance of recurrent gliomas are associated with *IDH1* mutation and TMZ-induced mutagenesis [44]. These results consistently suggest that the heterogeneous mechanisms at genomic level might be involved in the TMZ resistance during glioblastoma recurrence. Further analysis to integrate genomic mutations and transcriptomic reprogramming might be needed in near future.

In summary, we suggest that there are two different modes of transcriptomic reprograming during tumor recurrence, which could be predicted by the subtype classifiers. One is the sustained expression of stemness genes in the recurrent tumors, and the other is the transcriptomic reprograming to express neuron-like and drug resistance-related traits. Our integrative analysis could provide new insights on the transcriptomic reprogramming of recurrent glioblastomas, suggesting that different strategies might be required to overcome the subtype-dependent TMZ resistance.

## Supporting Information

S1 Fig Prediction of clinical outcomes by the subtype classifiers for G1 and G2 in independent data sets. (DOC)

S2 Fig Subtype prediction by unsupervised clustering of integrated data with TCGA data set. (DOC)

S3 Fig Differential expression of DNA repair genes between G1 and G2 subtypes. (DOC)

S4 Fig Comparison of *MGMT* and *MSH* expression in TCGA subtypes. (DOC)

S5 Fig Comparison of *ABCC1*, *ABCC 3*, *and ABCC 5* expression in G1 and G2 subtypes. (DOC)

S1 Table List of gene classifiers for G1 and G2 subtype. (DOC)

S2 Table Classification of recurrent glioblastomas based on the gene expression changes from the paired primary tumors. (DOC)

S3 Table Prediction of glioma subtypes by NTP. (DOC)

S4 Table Mutations of drug resistance-related genes in TCGA data. (DOC)
