# Supplementary material for: Recurrent Glioblastomas Reveal Molecular Subtypes Associated with Mechanistic Implications of Drug-Resistance
Source: PLoS One. 2015 Oct 14;10(10):e0140528. doi: 10.1371/journal.pone.0140528 (PMC4605710; doi:10.1371/journal.pone.0140528)
Supplement: S1 Fig — (DOC) [file pone.0140528.s001.doc]

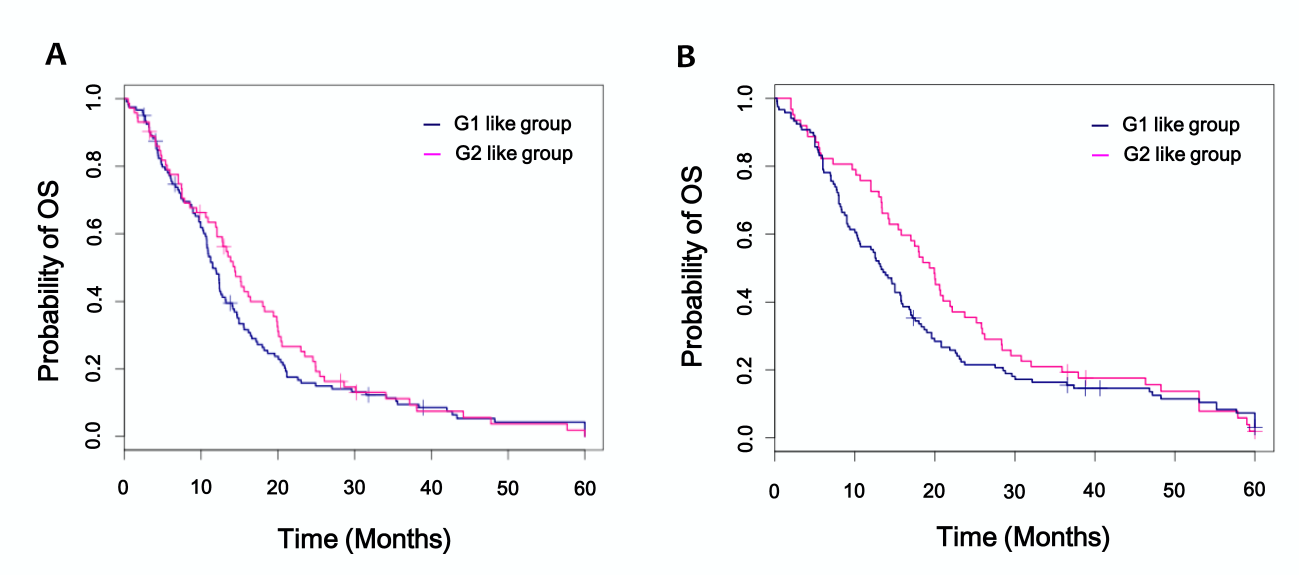


### S1 Figure. Prediction of clinical outcomes by the expression of G1 and G2 subtype classifiers in independent data sets

(**A-B**)Kaplan-Meir survival estimates of overall survival (**0S**) in the **REMBRANDT** (**A**) and **TCGA**(**B**) data set after subdivision into two groups based on the clustering result using the G1 and G2 signature of **Figure 2C** and **2D**, respectively. The groups with enriched expression of G1 and G2 signature were indicated with different colors.
