# Supplementary material for: Recurrent Glioblastomas Reveal Molecular Subtypes Associated with Mechanistic Implications of Drug-Resistance
Source: PLoS One. 2015 Oct 14;10(10):e0140528. doi: 10.1371/journal.pone.0140528 (PMC4605710; doi:10.1371/journal.pone.0140528)
Supplement: S2 Fig — (DOC) [file pone.0140528.s002.doc]

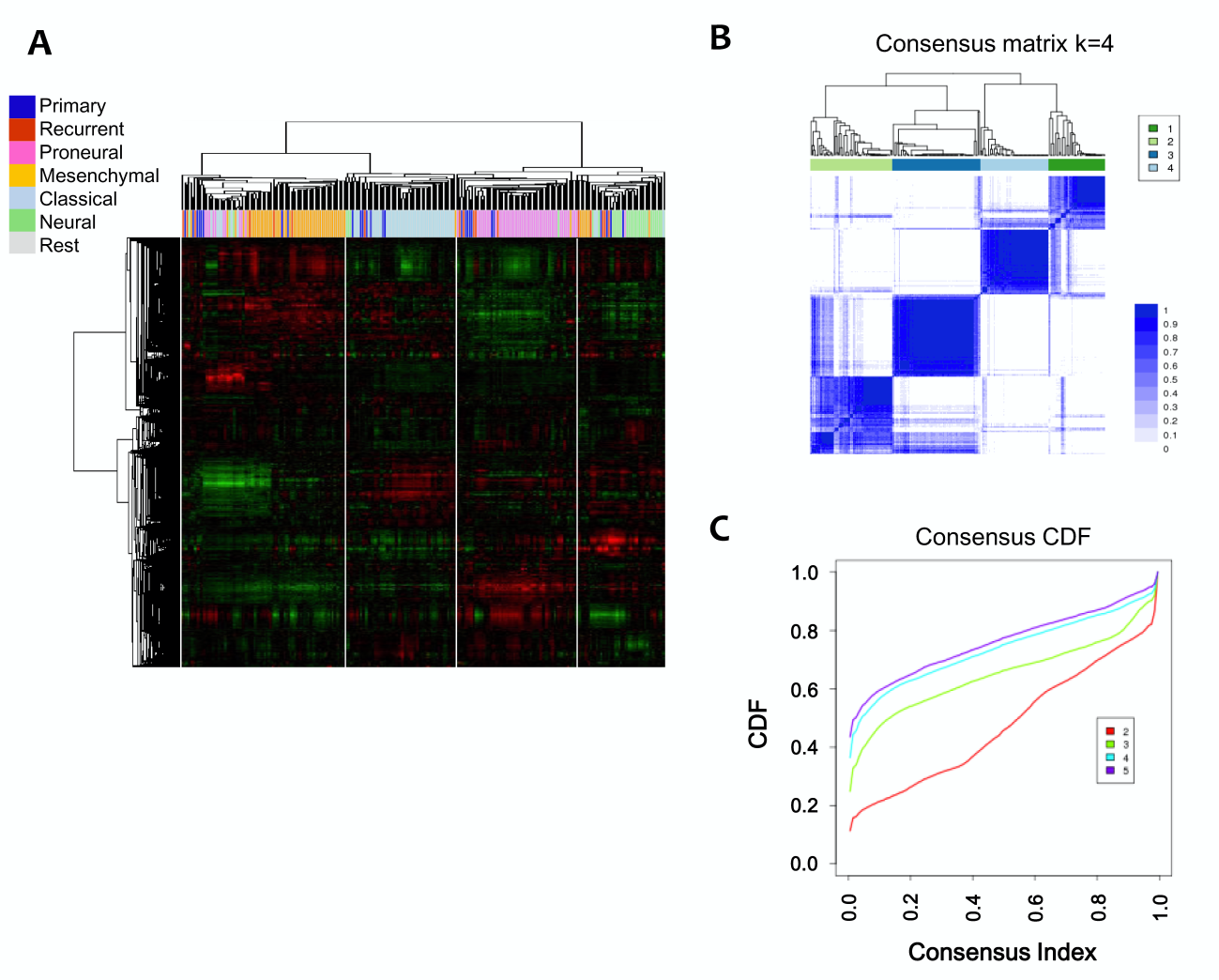


### S2 Figure . Subtype prediction by unsupervised clustering of integrated data with TCGA data set

(**A**) For subtype prediction, we performed unsupervised hierarchical clustering in the integrated dataset with **TCGA** 197 GBMs. The subtypes were marked with key colors corresponding to their subtypes, Proneuronal, Mesenchymal, Classical, and Neural type, based on the pre-defined **TCGA** phenotype information. The primary and recurrent tumors of our data are indicated with different colors. (**B**) Heatmap shows the consistency of the consensus clustering analysis with k=4. (**C**) Cumulative distribution functions (**CDF**) of the consensus matrix for each number k of clusters (k=2, 3, 4, 5) are plotted.
