# Supplementary material for: Recurrent Glioblastomas Reveal Molecular Subtypes Associated with Mechanistic Implications of Drug-Resistance
Source: PLoS One. 2015 Oct 14;10(10):e0140528. doi: 10.1371/journal.pone.0140528 (PMC4605710; doi:10.1371/journal.pone.0140528)
Supplement: S3 Fig — (DOC) [file pone.0140528.s003.doc]

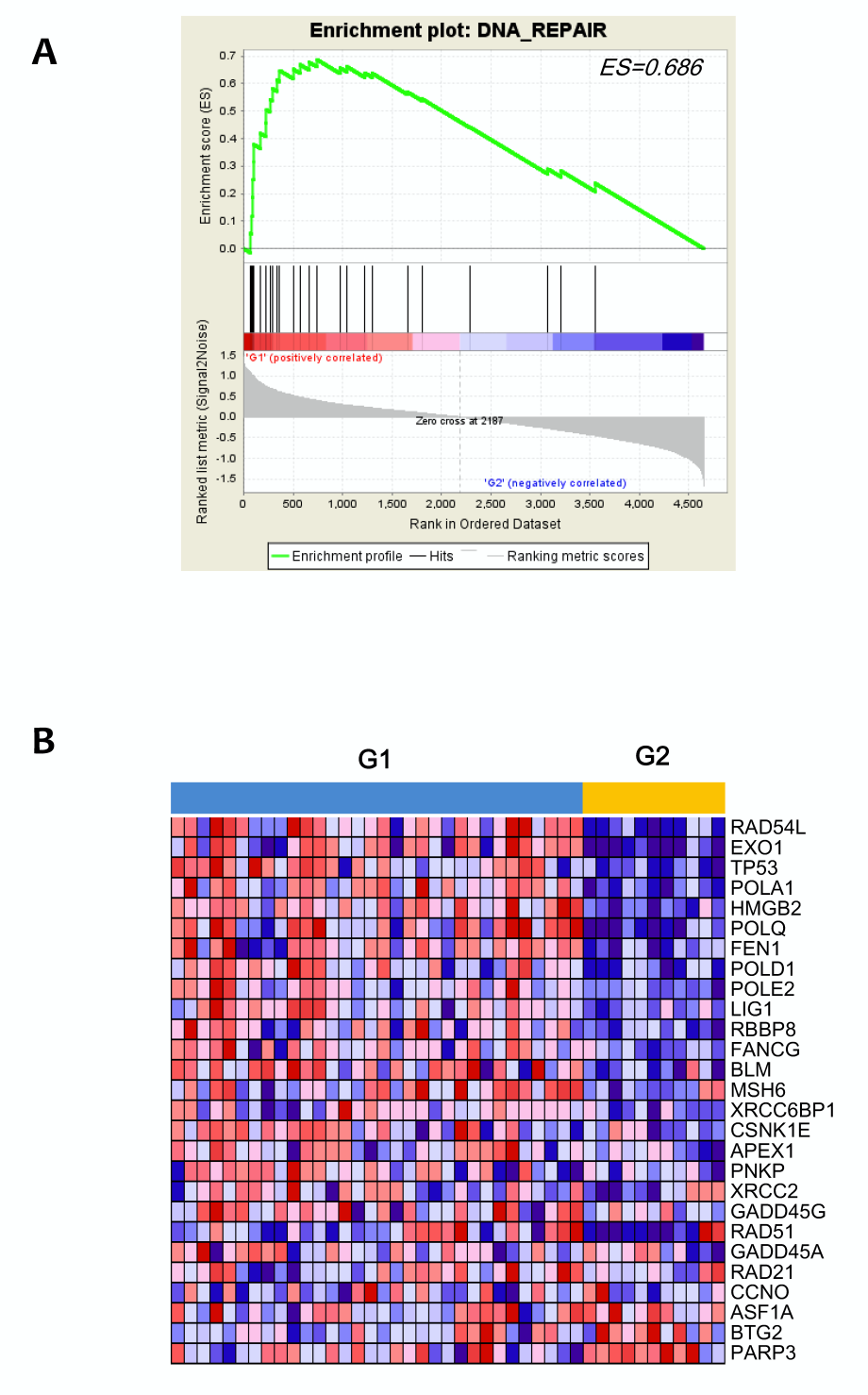


### S3 Figure. Differential expression of DNA repair genes between G1 and G2 subtypes

**(A**) The GSEA result shows the enrichment of the DNA_REPAIR signature. (**B**) The expression of the DNA_REPAIR genes was shown significantly down-regulated in G2 cluster, while up-regulated in G1 cluster
