# Supplementary material for: Recurrent Glioblastomas Reveal Molecular Subtypes Associated with Mechanistic Implications of Drug-Resistance
Source: PLoS One. 2015 Oct 14;10(10):e0140528. doi: 10.1371/journal.pone.0140528 (PMC4605710; doi:10.1371/journal.pone.0140528)
Supplement: S4 Fig — (DOC) [file pone.0140528.s004.doc]

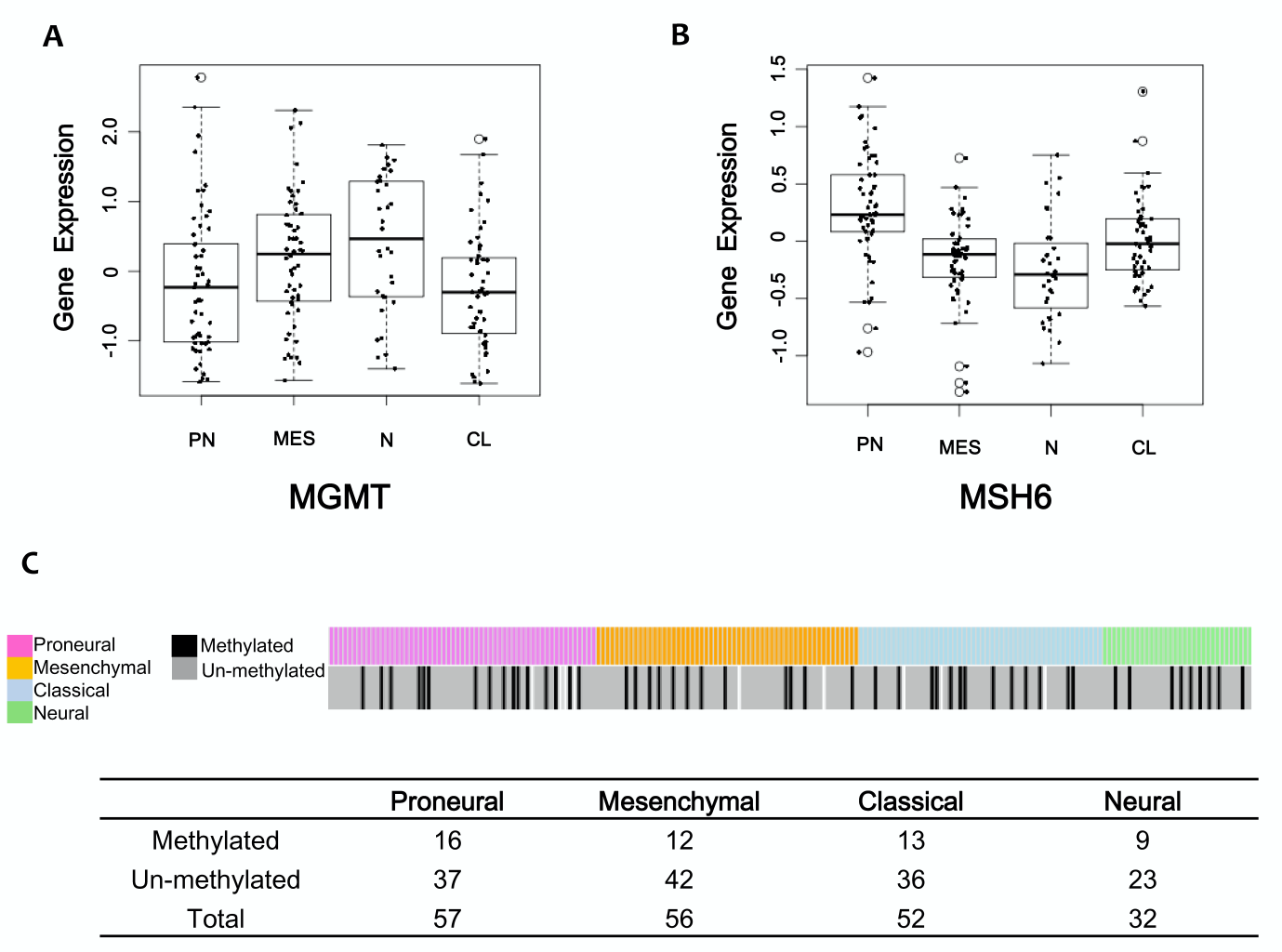


### S4 Figure. Comparison of *MGMT* and *MSH* expression in TCGA subtypes

(**A-B**)The expressions of *MGMT* (**A**) and *MSH6* (**B**) were evaluated in **TCGA** dataset. The subtypes were indicated corresponding to their subtypes, Proneuronal (**PN**), Mesenchymal(**MES**), Classical(**CL**), and Neural type(**N**), based on the pre-defined **TCGA** phenotype information. (**C**) The methylation status of the *MGMT* promoter in each subtype was plotted based on the pre-defined TCGA phenotype information. The number of incidence corresponding to the *MGMT* promoter methylation in each subtype were indicated in each table under the bar plots.
