# Supplementary material for: Recurrent Glioblastomas Reveal Molecular Subtypes Associated with Mechanistic Implications of Drug-Resistance
Source: PLoS One. 2015 Oct 14;10(10):e0140528. doi: 10.1371/journal.pone.0140528 (PMC4605710; doi:10.1371/journal.pone.0140528)
Supplement: S5 Fig — (DOC) [file pone.0140528.s005.doc]

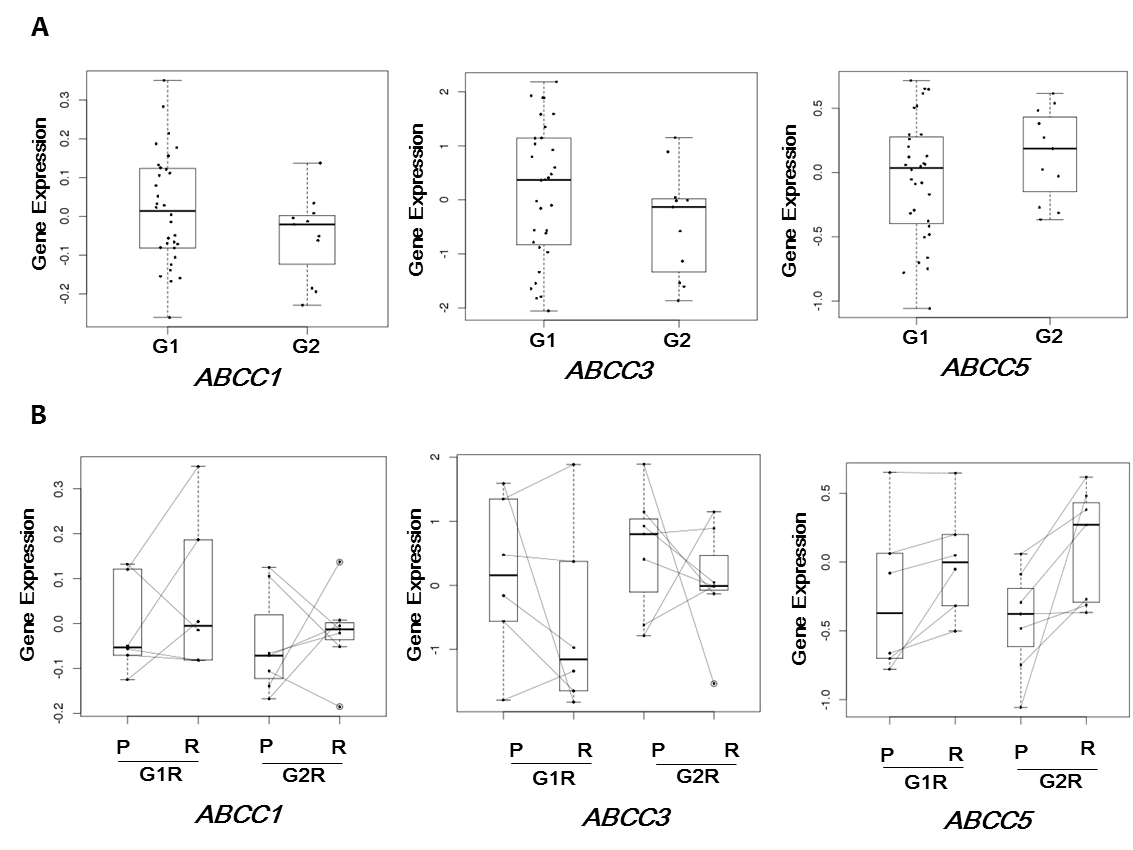


### S5 Figure. Comparison of *ABCC1, ABCC 3, and ABCC 5* expression in G1 and G2 subtypes

(A) The expressions of ABCC1 (left), ABCC3 (middle), and ABCC5 (right) were evaluated in G1 and G2 tumors. (B) The expressions of ABCC1 (left), ABCC3 (middle), and ABCC5 (right) were evaluated in primary and recurrent tumors of G1R and G2R pairs. P and R indicate primary and recurrent tumors, respectively. Lines were plotted to trace the expression changes between primary and recurrent tumors in paired group.
