# Supplementary material for: Recurrent Glioblastomas Reveal Molecular Subtypes Associated with Mechanistic Implications of Drug-Resistance
Source: PLoS One. 2015 Oct 14;10(10):e0140528. doi: 10.1371/journal.pone.0140528 (PMC4605710; doi:10.1371/journal.pone.0140528)
Supplement: S1 Table — (DOC) [file pone.0140528.s006.doc]

### S1 Table. List of gene classifiers for G1 and G2 subtype

| **Gene Symbol** | **T-statistic** | **P-value** | **Permuted P-value** | **FDR*** | **G1(average)** | **G2(average)** | **Fold Change** | **Subtype** |
| --- | --- | --- | --- | --- | --- | --- | --- | --- |
| FBL | -6.23508 | 1.12E-06 | 5.65E-05 | 0.002406 | -0.74496 | 0.25608 | -1.00104 | G1 |
| KIF11 | -7.45601 | 3.90E-09 | 9.86E-05 | 0.003305 | -0.74503 | 0.256104 | -1.00113 | G1 |
| MCM4 | -5.23979 | 1.04E-05 | 0.000558 | 0.009553 | -0.74564 | 0.256314 | -1.00196 | G1 |
| CDK1 | -7.64202 | 2.61E-09 | 0.000101 | 0.00334 | -0.74899 | 0.257465 | -1.00645 | G1 |
| SMC4 | -6.54729 | 6.11E-07 | 2.80E-05 | 0.001683 | -0.7505 | 0.257986 | -1.00849 | G1 |
| ECT2 | -5.66762 | 1.69E-06 | 0.000551 | 0.009471 | -0.75193 | 0.258477 | -1.01041 | G1 |
| CDCA3 | -7.2612 | 7.50E-09 | 8.27E-05 | 0.002967 | -0.75652 | 0.260053 | -1.01657 | G1 |
| BZW2 | -6.20507 | 6.72E-07 | 0.000104 | 0.003391 | -0.76138 | 0.261726 | -1.02311 | G1 |
| RAD51AP1 | -6.72188 | 4.35E-08 | 0.000174 | 0.004606 | -0.76226 | 0.262027 | -1.02429 | G1 |
| FBXO5 | -7.89887 | 2.62E-09 | 1.37E-05 | 0.001175 | -0.76448 | 0.262789 | -1.02727 | G1 |
| LDHA | -4.11731 | 0.001016 | 0.000124 | 0.003808 | -0.76475 | 0.262882 | -1.02763 | G1 |
| CCNB1 | -7.58131 | 2.55E-09 | 6.13E-05 | 0.00252 | -0.77379 | 0.26599 | -1.03978 | G1 |
| CCNF | -6.58353 | 9.74E-08 | 0.000126 | 0.003822 | -0.77613 | 0.266796 | -1.04293 | G1 |
| TROAP | -7.39856 | 1.02E-08 | 3.02E-05 | 0.001713 | -0.77773 | 0.267346 | -1.04508 | G1 |
| CDK2 | -7.33553 | 1.11E-07 | 6.80E-06 | 0.000786 | -0.7785 | 0.267608 | -1.0461 | G1 |
| CENPE | -7.28151 | 6.81E-09 | 0.000124 | 0.003808 | -0.77953 | 0.267965 | -1.0475 | G1 |
| POC1A | -6.83259 | 3.40E-08 | 0.000121 | 0.003751 | -0.77994 | 0.268104 | -1.04804 | G1 |
| TSKU | -4.93016 | 2.56E-05 | 0.000949 | 0.013423 | -0.78384 | 0.269445 | -1.05329 | G1 |
| CENPM | -7.42221 | 5.00E-09 | 5.45E-05 | 0.002369 | -0.78685 | 0.27048 | -1.05733 | G1 |
| FANCI | -6.75939 | 4.82E-08 | 0.000116 | 0.003636 | -0.78907 | 0.271244 | -1.06032 | G1 |
| MCM7 | -5.38332 | 9.02E-06 | 0.000311 | 0.006592 | -0.7923 | 0.272354 | -1.06465 | G1 |
| HNRNPH1 | -5.23018 | 3.83E-05 | 9.78E-05 | 0.00329 | -0.79648 | 0.27379 | -1.07027 | G1 |
| KIF14 | -7.83881 | 2.79E-09 | 1.61E-05 | 0.001262 | -0.80546 | 0.276878 | -1.08234 | G1 |
| FOXM1 | -7.18763 | 1.96E-08 | 4.04E-05 | 0.001998 | -0.8101 | 0.278473 | -1.08858 | G1 |
| LIMA1 | -4.47737 | 0.000177 | 0.000703 | 0.011238 | -0.81367 | 0.279701 | -1.09338 | G1 |
| LMNB2 | -6.56339 | 1.18E-06 | 1.43E-05 | 0.001197 | -0.81381 | 0.279749 | -1.09356 | G1 |
| FAM46A | -4.48442 | 0.000417 | 8.91E-05 | 0.003107 | -0.81677 | 0.280765 | -1.09753 | G1 |
| PLK4 | -8.01565 | 6.62E-10 | 4.70E-05 | 0.002177 | -0.81786 | 0.281141 | -1.099 | G1 |
| MAD2L1 | -7.31483 | 6.33E-09 | 0.000128 | 0.003834 | -0.82192 | 0.282536 | -1.10446 | G1 |
| FAM83D | -6.16745 | 2.58E-07 | 0.000643 | 0.010505 | -0.83072 | 0.285562 | -1.11629 | G1 |
| ATP6V1B1 | -4.75068 | 7.90E-05 | 0.000505 | 0.008918 | -0.85144 | 0.292684 | -1.14413 | G1 |
| FAM60A | -6.5623 | 5.14E-07 | 3.08E-05 | 0.001725 | -0.85209 | 0.292908 | -1.145 | G1 |
| CEP55 | -7.50726 | 4.23E-09 | 4.26E-05 | 0.002046 | -0.86081 | 0.295904 | -1.15672 | G1 |
| HMMR | -8.75105 | 8.51E-11 | 2.39E-05 | 0.001573 | -0.88044 | 0.302653 | -1.1831 | G1 |
| UBE2T | -5.7888 | 1.00E-06 | 0.000565 | 0.009613 | -0.88316 | 0.303587 | -1.18675 | G1 |
| ASF1B | -7.16399 | 2.09E-08 | 4.19E-05 | 0.002031 | -0.88731 | 0.305011 | -1.19232 | G1 |
| CENPA | -7.47946 | 3.55E-09 | 8.95E-05 | 0.003114 | -0.89176 | 0.306544 | -1.19831 | G1 |
| TACC3 | -5.56609 | 2.11E-06 | 0.000762 | 0.011734 | -0.89436 | 0.307435 | -1.20179 | G1 |
| KIF4A | -7.95293 | 8.03E-10 | 3.39E-05 | 0.001794 | -0.89957 | 0.309227 | -1.2088 | G1 |
| CPXM1 | -4.89515 | 5.17E-05 | 0.000417 | 0.007949 | -0.90378 | 0.310673 | -1.21445 | G1 |
| TTK | -7.81054 | 1.31E-09 | 3.78E-05 | 0.001913 | -0.90616 | 0.311494 | -1.21766 | G1 |
| RRM2 | -6.50416 | 8.84E-08 | 0.00042 | 0.007974 | -0.90619 | 0.311505 | -1.2177 | G1 |
| EZH2 | -6.42885 | 1.09E-07 | 0.000295 | 0.006361 | -0.90668 | 0.311671 | -1.21835 | G1 |
| KLHDC8A | -4.80227 | 4.40E-05 | 0.000918 | 0.013133 | -0.91479 | 0.314459 | -1.22925 | G1 |
| IGF2BP2 | -5.90309 | 5.94E-07 | 0.000785 | 0.01193 | -0.92141 | 0.316734 | -1.23814 | G1 |
| CDCA8 | -7.12134 | 2.76E-08 | 3.95E-05 | 0.001973 | -0.92507 | 0.317992 | -1.24306 | G1 |
| CDKN3 | -6.73798 | 8.95E-08 | 6.83E-05 | 0.002692 | -0.92698 | 0.31865 | -1.24563 | G1 |
| HSPA1B | -5.70748 | 1.23E-06 | 0.000717 | 0.011341 | -0.93199 | 0.320373 | -1.25237 | G1 |
| BUB1 | -7.9528 | 9.77E-10 | 2.50E-05 | 0.001589 | -0.93416 | 0.321117 | -1.25528 | G1 |
| GINS2 | -5.79384 | 1.89E-06 | 0.000242 | 0.005661 | -0.96014 | 0.330049 | -1.29019 | G1 |
| TYMS | -5.61869 | 2.12E-06 | 0.000536 | 0.00929 | -0.96038 | 0.330132 | -1.29051 | G1 |
| DLGAP5 | -7.59172 | 4.09E-09 | 3.02E-05 | 0.001713 | -0.96891 | 0.333062 | -1.30197 | G1 |
| SPC24 | -4.70468 | 0.000157 | 0.000193 | 0.004897 | -0.98373 | 0.338157 | -1.32189 | G1 |
| TRIP13 | -8.30078 | 3.04E-10 | 1.75E-05 | 0.001308 | -0.98573 | 0.338843 | -1.32457 | G1 |
| RND3 | -4.51196 | 0.000167 | 0.000624 | 0.01033 | -0.99676 | 0.342636 | -1.3394 | G1 |
| KIF2C | -7.72716 | 4.56E-09 | 1.68E-05 | 0.001277 | -0.999 | 0.343406 | -1.34241 | G1 |
| STK32B | -6.40925 | 3.37E-07 | 8.26E-05 | 0.002967 | -1.01601 | 0.349254 | -1.36526 | G1 |
| COL6A1 | -5.15739 | 1.16E-05 | 0.000764 | 0.011755 | -1.01678 | 0.34952 | -1.3663 | G1 |
| MCM2 | -6.522 | 2.03E-07 | 8.14E-05 | 0.002952 | -1.01771 | 0.349837 | -1.36754 | G1 |
| UHRF1 | -4.74745 | 0.000216 | 6.98E-05 | 0.002719 | -1.03998 | 0.357494 | -1.39748 | G1 |
| COL5A2 | -4.09999 | 0.000585 | 0.000817 | 0.012263 | -1.04952 | 0.360772 | -1.41029 | G1 |
| CCNA2 | -8.67429 | 9.00E-11 | 2.21E-05 | 0.00152 | -1.05133 | 0.361393 | -1.41272 | G1 |
| AURKA | -7.11519 | 1.73E-08 | 6.14E-05 | 0.00252 | -1.05297 | 0.361958 | -1.41492 | G1 |
| WEE1 | -7.88722 | 3.09E-08 | 3.06E-06 | 0.000545 | -1.07433 | 0.369299 | -1.44362 | G1 |
| TRIB2 | -5.05863 | 1.84E-05 | 0.000717 | 0.011341 | -1.07602 | 0.369881 | -1.4459 | G1 |
| E2F2 | -4.92886 | 2.79E-05 | 0.000847 | 0.012496 | -1.09906 | 0.377803 | -1.47686 | G1 |
| BIRC5 | -7.79416 | 1.71E-09 | 2.89E-05 | 0.001697 | -1.12416 | 0.38643 | -1.51059 | G1 |
| NT5DC2 | -6.51366 | 2.35E-06 | 8.64E-06 | 0.000884 | -1.12472 | 0.386623 | -1.51134 | G1 |
| MELK | -7.50191 | 8.71E-09 | 2.32E-05 | 0.001546 | -1.16723 | 0.401234 | -1.56846 | G1 |
| KIFC1 | -7.58034 | 5.25E-09 | 2.56E-05 | 0.001595 | -1.16981 | 0.402121 | -1.57193 | G1 |
| HJURP | -8.30964 | 1.31E-09 | 5.91E-06 | 0.000735 | -1.17345 | 0.403373 | -1.57682 | G1 |
| KIF20A | -8.76107 | 9.30E-11 | 7.83E-06 | 0.000843 | -1.17401 | 0.403565 | -1.57757 | G1 |
| TPX2 | -7.71339 | 4.68E-09 | 1.73E-05 | 0.001293 | -1.17718 | 0.404656 | -1.58184 | G1 |
| AURKB | -7.28055 | 2.41E-08 | 2.43E-05 | 0.001583 | -1.18104 | 0.405984 | -1.58703 | G1 |
| CKAP2L | -8.22515 | 7.89E-10 | 1.04E-05 | 0.000984 | -1.20283 | 0.413473 | -1.61631 | G1 |
| CDCA5 | -7.57466 | 9.84E-09 | 1.65E-05 | 0.00127 | -1.25457 | 0.43126 | -1.68583 | G1 |
| PBK | -6.37441 | 4.60E-07 | 7.20E-05 | 0.00276 | -1.25508 | 0.431432 | -1.68651 | G1 |
| CKS2 | -6.67916 | 4.03E-07 | 2.43E-05 | 0.001583 | -1.25765 | 0.432318 | -1.68997 | G1 |
| CDC45 | -7.15022 | 2.12E-08 | 4.39E-05 | 0.002089 | -1.26065 | 0.43335 | -1.694 | G1 |
| PRC1 | -7.65442 | 1.31E-08 | 1.05E-05 | 0.000984 | -1.26174 | 0.433723 | -1.69546 | G1 |
| FAM64A | -6.89757 | 2.58E-07 | 1.58E-05 | 0.001253 | -1.27881 | 0.439591 | -1.7184 | G1 |
| VEGFA | -5.02788 | 2.78E-05 | 0.000482 | 0.008688 | -1.29796 | 0.446173 | -1.74413 | G1 |
| NCAPG | -8.15081 | 7.47E-09 | 3.45E-06 | 0.000569 | -1.3079 | 0.449591 | -1.75749 | G1 |
| PTTG3P | -7.26801 | 1.18E-07 | 8.10E-06 | 0.000855 | -1.34806 | 0.463397 | -1.81146 | G1 |
| ASPM | -7.61441 | 4.66E-09 | 2.47E-05 | 0.001589 | -1.34884 | 0.463662 | -1.8125 | G1 |
| IGF2BP3 | -7.72865 | 8.69E-08 | 2.43E-06 | 0.0005 | -1.39236 | 0.478623 | -1.87098 | G1 |
| NUSAP1 | -7.94533 | 4.81E-09 | 8.05E-06 | 0.000854 | -1.39418 | 0.479249 | -1.87343 | G1 |
| CDC20 | -6.43133 | 4.65E-07 | 5.65E-05 | 0.002406 | -1.40674 | 0.483567 | -1.89031 | G1 |
| PTTG1 | -7.83223 | 4.22E-08 | 2.93E-06 | 0.000538 | -1.42358 | 0.489357 | -1.91294 | G1 |
| CENPF | -6.7687 | 1.62E-07 | 3.67E-05 | 0.001874 | -1.47186 | 0.505953 | -1.97782 | G1 |
| CCNB2 | -7.21237 | 7.68E-08 | 1.33E-05 | 0.001154 | -1.48572 | 0.510716 | -1.99643 | G1 |
| UBE2C | -7.43537 | 4.01E-08 | 1.01E-05 | 0.000975 | -1.51495 | 0.520763 | -2.03571 | G1 |
| IGFBP2 | -5.80365 | 2.07E-05 | 1.21E-05 | 0.001092 | -1.61352 | 0.554647 | -2.16817 | G1 |
| TOP2A | -6.35797 | 2.79E-06 | 1.35E-05 | 0.001167 | -1.7842 | 0.613317 | -2.39751 | G1 |
| NKX6-2 | 6.157672 | 8.50E-06 | 8.25E-06 | 0.000865 | 2.466746 | -0.84794 | 3.31469 | G2 |
| MAG | 6.245452 | 5.71E-06 | 9.40E-06 | 0.000938 | 2.382374 | -0.81894 | 3.201316 | G2 |
| MAL | 6.273059 | 6.22E-06 | 7.47E-06 | 0.000827 | 2.364513 | -0.8128 | 3.177315 | G2 |
| MOBP | 6.098143 | 1.81E-05 | 3.44E-06 | 0.000569 | 2.334985 | -0.80265 | 3.137636 | G2 |
| TF | 8.252171 | 4.40E-10 | 1.48E-05 | 0.001215 | 2.287455 | -0.78631 | 3.073768 | G2 |
| CARNS1 | 6.149815 | 2.42E-05 | 1.58E-06 | 0.000398 | 2.207805 | -0.75893 | 2.966738 | G2 |
| PTGDS | 8.345954 | 2.35E-10 | 3.07E-05 | 0.001725 | 2.082209 | -0.71576 | 2.797968 | G2 |
| MOG | 5.971119 | 2.38E-05 | 4.04E-06 | 0.000628 | 2.076294 | -0.71373 | 2.79002 | G2 |
| ENPP2 | 6.030095 | 1.38E-05 | 7.42E-06 | 0.000827 | 2.00683 | -0.68985 | 2.696678 | G2 |
| CNDP1 | 5.454926 | 6.37E-05 | 1.04E-05 | 0.000984 | 1.996977 | -0.68646 | 2.683438 | G2 |
| BCAS1 | 6.045309 | 8.58E-07 | 0.000168 | 0.004495 | 1.995223 | -0.68586 | 2.681081 | G2 |
| MBP | 4.984606 | 9.69E-05 | 8.22E-05 | 0.002966 | 1.99021 | -0.68413 | 2.674345 | G2 |
| GRM3 | 7.000797 | 4.18E-06 | 6.54E-07 | 0.000334 | 1.969422 | -0.67699 | 2.646411 | G2 |
| PLP1 | 5.993835 | 3.33E-06 | 5.29E-05 | 0.00233 | 1.96841 | -0.67664 | 2.645051 | G2 |
| TMEM125 | 5.744676 | 7.43E-05 | 1.21E-06 | 0.000377 | 1.964602 | -0.67533 | 2.639934 | G2 |
| KLK6 | 5.628101 | 3.50E-05 | 1.25E-05 | 0.001121 | 1.891942 | -0.65035 | 2.542297 | G2 |
| TMEM144 | 5.534522 | 4.13E-05 | 1.56E-05 | 0.001246 | 1.869428 | -0.64262 | 2.512044 | G2 |
| LHPP | 7.211895 | 1.23E-06 | 1.30E-06 | 0.000377 | 1.837659 | -0.6317 | 2.469354 | G2 |
| ETNPPL | 6.66024 | 9.34E-06 | 7.87E-07 | 0.000348 | 1.828336 | -0.62849 | 2.456827 | G2 |
| PACSIN1 | 4.170513 | 0.001438 | 1.13E-05 | 0.001031 | 1.811155 | -0.62258 | 2.433739 | G2 |
| SH3GL3 | 5.752739 | 3.65E-05 | 5.80E-06 | 0.000733 | 1.786796 | -0.61421 | 2.401007 | G2 |
| SEPT4 | 8.313725 | 1.81E-07 | 2.84E-07 | 0.000317 | 1.772261 | -0.60921 | 2.381476 | G2 |
| CNTN2 | 4.768883 | 0.000157 | 0.000125 | 0.003822 | 1.748172 | -0.60093 | 2.349106 | G2 |
| PPP1R14A | 5.579427 | 5.52E-05 | 6.72E-06 | 0.000784 | 1.746718 | -0.60043 | 2.347152 | G2 |
| SEC14L5 | 6.916101 | 1.24E-05 | 1.66E-07 | 0.000317 | 1.729447 | -0.5945 | 2.323944 | G2 |
| EFHD1 | 8.237582 | 4.72E-10 | 1.47E-05 | 0.001215 | 1.726285 | -0.59341 | 2.319695 | G2 |
| HHATL | 6.088838 | 2.30E-05 | 2.37E-06 | 0.0005 | 1.72125 | -0.59168 | 2.312929 | G2 |
| ADAP1 | 7.385111 | 2.68E-07 | 2.96E-06 | 0.000538 | 1.701634 | -0.58494 | 2.286571 | G2 |
| GJC2 | 6.221054 | 2.28E-05 | 1.24E-06 | 0.000377 | 1.690519 | -0.58112 | 2.271635 | G2 |
| PI16 | 4.945201 | 0.000192 | 2.18E-05 | 0.001512 | 1.684719 | -0.57912 | 2.263841 | G2 |
| NINJ2 | 6.228742 | 2.20E-05 | 1.28E-06 | 0.000377 | 1.65297 | -0.56821 | 2.221179 | G2 |
| CNTNAP2 | 5.728143 | 2.87E-05 | 1.04E-05 | 0.000984 | 1.636141 | -0.56242 | 2.198564 | G2 |
| OPALIN | 4.235259 | 0.001661 | 1.39E-06 | 0.000394 | 1.62095 | -0.5572 | 2.178152 | G2 |
| CPNE6 | 5.357399 | 0.000219 | 5.39E-07 | 0.000319 | 1.609469 | -0.55325 | 2.162724 | G2 |
| CAPN3 | 6.140267 | 1.91E-05 | 2.55E-06 | 0.000519 | 1.607229 | -0.55248 | 2.159714 | G2 |
| NKAIN2 | 5.78982 | 3.75E-05 | 4.49E-06 | 0.000662 | 1.600515 | -0.55018 | 2.150692 | G2 |
| SLC39A12 | 8.364784 | 1.00E-07 | 3.90E-07 | 0.000317 | 1.599787 | -0.54993 | 2.149714 | G2 |
| AKR1C3 | 6.653789 | 2.37E-07 | 4.18E-05 | 0.002031 | 1.593827 | -0.54788 | 2.141705 | G2 |
| SH3GL2 | 5.817507 | 5.23E-06 | 7.11E-05 | 0.002738 | 1.572479 | -0.54054 | 2.113019 | G2 |
| BRINP1 | 5.311546 | 3.74E-05 | 6.46E-05 | 0.002589 | 1.556294 | -0.53498 | 2.09127 | G2 |
| PLLP | 5.85497 | 1.65E-05 | 1.33E-05 | 0.001154 | 1.553672 | -0.53407 | 2.087747 | G2 |
| TPD52L1 | 8.472286 | 1.72E-07 | 1.99E-07 | 0.000317 | 1.538795 | -0.52896 | 2.067755 | G2 |
| FAIM2 | 6.806149 | 6.54E-07 | 9.96E-06 | 0.000967 | 1.516035 | -0.52114 | 2.037172 | G2 |
| CNTNAP4 | 6.044064 | 5.00E-05 | 5.65E-07 | 0.000319 | 1.486216 | -0.51089 | 1.997102 | G2 |
| SNAP91 | 5.634328 | 4.34E-06 | 0.000209 | 0.005161 | 1.483612 | -0.50999 | 1.993604 | G2 |
| CALY | 3.980123 | 0.001771 | 4.18E-05 | 0.002031 | 1.4797 | -0.50865 | 1.988347 | G2 |
| CLDN11 | 4.274208 | 0.00036 | 0.000679 | 0.010937 | 1.470541 | -0.5055 | 1.976039 | G2 |
| PLEKHH1 | 6.528214 | 1.51E-05 | 6.25E-07 | 0.000334 | 1.469548 | -0.50516 | 1.974705 | G2 |
| CA4 | 5.17481 | 0.000153 | 7.72E-06 | 0.000842 | 1.454542 | -0.5 | 1.954541 | G2 |
| OMG | 8.400998 | 1.88E-10 | 2.26E-05 | 0.001531 | 1.4534 | -0.49961 | 1.953007 | G2 |
| ERMN | 5.145309 | 0.000275 | 1.30E-06 | 0.000377 | 1.44913 | -0.49814 | 1.947269 | G2 |
| SERPINI1 | 7.26976 | 3.91E-06 | 2.69E-07 | 0.000317 | 1.441216 | -0.49542 | 1.936634 | G2 |
| APLNR | 4.837221 | 7.98E-05 | 0.000293 | 0.006323 | 1.439412 | -0.4948 | 1.93421 | G2 |
| MAP7 | 6.474462 | 9.79E-06 | 1.63E-06 | 0.000398 | 1.431316 | -0.49201 | 1.92333 | G2 |
| FAM107A | 6.805841 | 3.11E-08 | 0.000217 | 0.005309 | 1.429416 | -0.49136 | 1.920777 | G2 |
| PIP4K2A | 5.764625 | 3.59E-05 | 5.62E-06 | 0.000721 | 1.417457 | -0.48725 | 1.904708 | G2 |
| RCAN2 | 6.060599 | 1.88E-06 | 7.16E-05 | 0.002749 | 1.412261 | -0.48546 | 1.897725 | G2 |
| HSPB8 | 6.291476 | 1.27E-06 | 3.96E-05 | 0.001973 | 1.408965 | -0.48433 | 1.893297 | G2 |
| SLC31A2 | 5.335879 | 0.000114 | 5.51E-06 | 0.000721 | 1.40648 | -0.48348 | 1.889957 | G2 |
| PPP1R16B | 6.42068 | 1.76E-05 | 7.73E-07 | 0.000348 | 1.400642 | -0.48147 | 1.882113 | G2 |
| SPOCK1 | 6.211307 | 1.21E-06 | 5.78E-05 | 0.002442 | 1.395797 | -0.47981 | 1.875602 | G2 |
| DAAM2 | 6.127415 | 6.81E-06 | 1.27E-05 | 0.001126 | 1.387754 | -0.47704 | 1.864794 | G2 |
| S1PR5 | 4.915232 | 0.000375 | 2.82E-06 | 0.000535 | 1.385716 | -0.47634 | 1.862055 | G2 |
| HSPA2 | 5.162259 | 8.53E-05 | 3.45E-05 | 0.001806 | 1.382502 | -0.47523 | 1.857737 | G2 |
| SLC17A7 | 3.517242 | 0.004518 | 7.53E-05 | 0.002822 | 1.363549 | -0.46872 | 1.832269 | G2 |
| TPPP | 6.036448 | 3.52E-05 | 1.33E-06 | 0.000381 | 1.363522 | -0.46871 | 1.832233 | G2 |
| PAQR8 | 7.630606 | 2.55E-07 | 1.44E-06 | 0.000396 | 1.358998 | -0.46716 | 1.826154 | G2 |
| CNP | 5.494289 | 0.000122 | 1.54E-06 | 0.000398 | 1.358699 | -0.46705 | 1.825751 | G2 |
| TMEM151A | 6.052812 | 5.08E-05 | 5.17E-07 | 0.000319 | 1.349629 | -0.46394 | 1.813564 | G2 |
| NRGN | 3.549931 | 0.003487 | 0.000289 | 0.006261 | 1.338246 | -0.46002 | 1.798268 | G2 |
| PLA2G4C | 9.245824 | 1.46E-10 | 1.61E-06 | 0.000398 | 1.326273 | -0.45591 | 1.782179 | G2 |
| RAPGEF5 | 5.484 | 4.90E-05 | 1.50E-05 | 0.001218 | 1.324917 | -0.45544 | 1.780357 | G2 |
| VSNL1 | 3.342829 | 0.006053 | 0.000157 | 0.004288 | 1.319101 | -0.45344 | 1.772543 | G2 |
| PRKCB | 6.05569 | 2.82E-06 | 4.82E-05 | 0.002198 | 1.315209 | -0.4521 | 1.767312 | G2 |
| KIAA1598 | 5.658303 | 2.24E-05 | 2.25E-05 | 0.00153 | 1.313202 | -0.45141 | 1.764616 | G2 |
| PLEKHB1 | 5.824921 | 2.62E-06 | 0.000147 | 0.004131 | 1.302495 | -0.44773 | 1.750227 | G2 |
| ALDH1A1 | 5.414895 | 3.93E-05 | 3.33E-05 | 0.001788 | 1.273137 | -0.43764 | 1.710778 | G2 |
| AMER2 | 5.321626 | 4.72E-05 | 4.11E-05 | 0.002017 | 1.266294 | -0.43529 | 1.701582 | G2 |
| CHD5 | 3.520963 | 0.004309 | 0.000105 | 0.003401 | 1.252321 | -0.43049 | 1.682806 | G2 |
| LGI3 | 6.342838 | 3.64E-05 | 2.43E-07 | 0.000317 | 1.245417 | -0.42811 | 1.67353 | G2 |
| GABRB1 | 4.574836 | 0.000114 | 0.000811 | 0.012193 | 1.243814 | -0.42756 | 1.671375 | G2 |
| DDN | 3.809015 | 0.002642 | 3.63E-05 | 0.001871 | 1.242066 | -0.42696 | 1.669027 | G2 |
| PAQR6 | 6.985895 | 8.25E-07 | 4.23E-06 | 0.000642 | 1.240471 | -0.42641 | 1.666883 | G2 |
| GPR62 | 4.907035 | 0.000487 | 8.68E-07 | 0.000353 | 1.240308 | -0.42636 | 1.666664 | G2 |
| ANKS1B | 5.84896 | 5.29E-06 | 6.07E-05 | 0.002507 | 1.23991 | -0.42622 | 1.666129 | G2 |
| KIF1A | 6.195761 | 2.46E-07 | 0.00036 | 0.007238 | 1.239346 | -0.42603 | 1.665372 | G2 |
| PPP2R2C | 5.850273 | 5.41E-05 | 1.43E-06 | 0.000396 | 1.237965 | -0.42555 | 1.663515 | G2 |
| SNCA | 6.044044 | 2.01E-05 | 3.77E-06 | 0.000604 | 1.232813 | -0.42378 | 1.656592 | G2 |
| SLC7A14 | 5.364538 | 5.06E-05 | 2.83E-05 | 0.001683 | 1.230709 | -0.42306 | 1.653765 | G2 |
| AK5 | 6.152049 | 7.42E-05 | 9.80E-08 | 0.000317 | 1.229981 | -0.42281 | 1.652787 | G2 |
| ARHGAP44 | 6.447665 | 2.45E-05 | 3.52E-07 | 0.000317 | 1.226538 | -0.42162 | 1.64816 | G2 |
| SCN2B | 7.645599 | 1.24E-06 | 3.15E-07 | 0.000317 | 1.223946 | -0.42073 | 1.644678 | G2 |
| DBNDD2 | 6.571436 | 6.21E-06 | 2.09E-06 | 0.000463 | 1.22223 | -0.42014 | 1.642371 | G2 |
| SYT1 | 3.142843 | 0.008287 | 0.000473 | 0.008597 | 1.212223 | -0.4167 | 1.628925 | G2 |
| CCK | 3.838366 | 0.002387 | 4.72E-05 | 0.002177 | 1.207517 | -0.41508 | 1.622602 | G2 |
| RAB40B | 5.882119 | 6.40E-05 | 7.72E-07 | 0.000348 | 1.207201 | -0.41498 | 1.622177 | G2 |
| NDRG2 | 4.292065 | 0.000354 | 0.000618 | 0.010265 | 1.206907 | -0.41487 | 1.621781 | G2 |
| PPP1R1B | 6.59341 | 5.82E-07 | 2.45E-05 | 0.001589 | 1.20019 | -0.41257 | 1.612756 | G2 |
| MAP6D1 | 4.875435 | 0.000259 | 1.57E-05 | 0.001249 | 1.199391 | -0.41229 | 1.611682 | G2 |
| HPCAL4 | 4.391313 | 0.000511 | 0.000106 | 0.00342 | 1.186546 | -0.40788 | 1.594422 | G2 |
| CYP2J2 | 6.627529 | 4.57E-07 | 2.65E-05 | 0.001616 | 1.184712 | -0.40724 | 1.591956 | G2 |
| NWD1 | 5.051315 | 2.82E-05 | 0.000415 | 0.007924 | 1.17935 | -0.4054 | 1.584752 | G2 |
| CHADL | 6.385514 | 6.51E-06 | 4.32E-06 | 0.000647 | 1.176299 | -0.40435 | 1.580652 | G2 |
| CTSH | 6.028437 | 3.56E-06 | 4.23E-05 | 0.002033 | 1.175708 | -0.40415 | 1.579857 | G2 |
| SLC12A5 | 3.731263 | 0.003058 | 4.27E-05 | 0.002047 | 1.172613 | -0.40309 | 1.575699 | G2 |
| PLCH2 | 5.31027 | 6.28E-05 | 2.58E-05 | 0.001595 | 1.171288 | -0.40263 | 1.573919 | G2 |
| TCEAL6 | 5.516013 | 4.51E-05 | 1.46E-05 | 0.001213 | 1.1712 | -0.4026 | 1.5738 | G2 |
| NIPAL3 | 4.923585 | 0.000175 | 3.29E-05 | 0.001788 | 1.165398 | -0.40061 | 1.566003 | G2 |
| SLC45A3 | 4.814511 | 0.000373 | 7.31E-06 | 0.000827 | 1.162774 | -0.3997 | 1.562477 | G2 |
| NECAB1 | 5.12954 | 0.00013 | 1.63E-05 | 0.001265 | 1.155519 | -0.39721 | 1.552729 | G2 |
| CYP46A1 | 6.602579 | 1.05E-06 | 1.38E-05 | 0.001175 | 1.153658 | -0.39657 | 1.550227 | G2 |
| SEPP1 | 4.434048 | 0.000267 | 0.000411 | 0.007888 | 1.152349 | -0.39612 | 1.54847 | G2 |
| KCNT1 | 3.434344 | 0.005053 | 0.000134 | 0.003935 | 1.145492 | -0.39376 | 1.539255 | G2 |
| CSRP1 | 4.369059 | 0.000477 | 0.000158 | 0.004303 | 1.144824 | -0.39353 | 1.538357 | G2 |
| DLG2 | 6.651527 | 1.64E-05 | 3.07E-07 | 0.000317 | 1.140656 | -0.3921 | 1.532756 | G2 |
| MYBPC1 | 4.262521 | 0.00048 | 0.000371 | 0.007365 | 1.13981 | -0.39181 | 1.531619 | G2 |
| BCYRN1 | 5.348067 | 1.97E-05 | 0.000137 | 0.003965 | 1.13799 | -0.39118 | 1.529174 | G2 |
| NEFM | 3.698695 | 0.003119 | 6.29E-05 | 0.002536 | 1.130603 | -0.38864 | 1.519248 | G2 |
| TMEM63A | 5.928247 | 5.53E-05 | 8.55E-07 | 0.000353 | 1.126191 | -0.38713 | 1.513319 | G2 |
| GPR37 | 4.110743 | 0.000679 | 0.000503 | 0.008909 | 1.120967 | -0.38533 | 1.506299 | G2 |
| NTSR2 | 5.659503 | 0.000132 | 3.67E-07 | 0.000317 | 1.120134 | -0.38505 | 1.505181 | G2 |
| SLC5A11 | 5.309044 | 0.000204 | 1.08E-06 | 0.000375 | 1.114982 | -0.38328 | 1.498257 | G2 |
| GRIN1 | 3.869593 | 0.00259 | 1.55E-05 | 0.001246 | 1.113651 | -0.38282 | 1.496468 | G2 |
| RASGRP3 | 4.913688 | 0.000288 | 7.97E-06 | 0.000849 | 1.112807 | -0.38253 | 1.495335 | G2 |
| PTPRD | 8.483098 | 4.90E-09 | 1.82E-06 | 0.000417 | 1.111251 | -0.38199 | 1.493243 | G2 |
| KCNK1 | 8.96452 | 3.19E-09 | 7.25E-07 | 0.000343 | 1.107701 | -0.38077 | 1.488473 | G2 |
| ABCA2 | 5.112196 | 0.000181 | 7.45E-06 | 0.000827 | 1.105395 | -0.37998 | 1.485375 | G2 |
| MGST1 | 5.704345 | 3.46E-06 | 0.000192 | 0.004885 | 1.097204 | -0.37716 | 1.474368 | G2 |
| SEMA3B | 4.942817 | 0.000185 | 2.47E-05 | 0.001589 | 1.096587 | -0.37695 | 1.473538 | G2 |
| ATP6V1G2 | 4.952422 | 8.38E-05 | 0.000133 | 0.003911 | 1.093631 | -0.37594 | 1.469566 | G2 |
| EPB41L3 | 5.192961 | 1.49E-05 | 0.000451 | 0.008369 | 1.09018 | -0.37475 | 1.46493 | G2 |
| PRRG1 | 5.274171 | 0.000126 | 6.55E-06 | 0.000779 | 1.087562 | -0.37385 | 1.461411 | G2 |
| ITPKB | 8.937411 | 3.95E-11 | 8.79E-06 | 0.00089 | 1.077184 | -0.37028 | 1.447466 | G2 |
| CACNG3 | 4.015071 | 0.001867 | 1.82E-05 | 0.001337 | 1.076203 | -0.36994 | 1.446148 | G2 |
| RAB6B | 7.062705 | 7.69E-07 | 3.45E-06 | 0.000569 | 1.075425 | -0.36968 | 1.445102 | G2 |
| GNG3 | 3.650456 | 0.002968 | 0.000182 | 0.004745 | 1.075073 | -0.36956 | 1.44463 | G2 |
| FBXO2 | 9.826162 | 6.14E-09 | 9.37E-08 | 0.000317 | 1.074951 | -0.36951 | 1.444465 | G2 |
| LARP6 | 6.61183 | 4.92E-06 | 2.42E-06 | 0.0005 | 1.072134 | -0.36855 | 1.44068 | G2 |
| DYNC1I1 | 5.654172 | 6.12E-06 | 0.000128 | 0.003834 | 1.071749 | -0.36841 | 1.440163 | G2 |
| RANBP3L | 6.661566 | 7.57E-06 | 1.08E-06 | 0.000375 | 1.068587 | -0.36733 | 1.435913 | G2 |
| CMTM5 | 5.212281 | 4.63E-05 | 7.96E-05 | 0.002917 | 1.067724 | -0.36703 | 1.434755 | G2 |
| BIN1 | 5.908466 | 4.80E-06 | 5.17E-05 | 0.002306 | 1.065993 | -0.36644 | 1.432428 | G2 |
| CLDND1 | 4.620558 | 0.000394 | 3.37E-05 | 0.001788 | 1.061158 | -0.36477 | 1.425931 | G2 |
| SYN2 | 3.538014 | 0.004517 | 5.06E-05 | 0.002272 | 1.060664 | -0.3646 | 1.425267 | G2 |
| LPAR1 | 4.261611 | 0.000361 | 0.000742 | 0.011548 | 1.059332 | -0.36415 | 1.423478 | G2 |
| BEST1 | 5.944048 | 1.39E-05 | 1.11E-05 | 0.001028 | 1.057043 | -0.36336 | 1.420402 | G2 |
| IFIT1 | 4.679859 | 0.000119 | 0.000388 | 0.007599 | 1.05677 | -0.36326 | 1.420035 | G2 |
| GPC5 | 5.773091 | 1.23E-05 | 3.03E-05 | 0.001713 | 1.046593 | -0.35977 | 1.406359 | G2 |
| GPIHBP1 | 4.259628 | 0.001266 | 7.58E-06 | 0.000834 | 1.044559 | -0.35907 | 1.403627 | G2 |
| GJB1 | 4.678537 | 0.000391 | 2.07E-05 | 0.001457 | 1.038462 | -0.35697 | 1.395433 | G2 |
| MAST3 | 8.014714 | 2.32E-06 | 5.00E-08 | 0.000317 | 1.038353 | -0.35693 | 1.395287 | G2 |
| PDK4 | 4.154764 | 0.000573 | 0.000553 | 0.009498 | 1.034675 | -0.35567 | 1.390345 | G2 |
| CLCA4 | 4.807762 | 0.000569 | 1.09E-06 | 0.000375 | 1.033361 | -0.35522 | 1.388578 | G2 |
| QDPR | 5.576146 | 6.53E-05 | 4.71E-06 | 0.00067 | 1.032375 | -0.35488 | 1.387254 | G2 |
| MAOB | 4.006048 | 0.000778 | 0.000833 | 0.01239 | 1.03141 | -0.35455 | 1.385958 | G2 |
| CA11 | 4.496096 | 0.000357 | 0.000127 | 0.003834 | 1.031109 | -0.35444 | 1.385553 | G2 |
| KCNK12 | 4.595532 | 0.000157 | 0.000401 | 0.00777 | 1.030921 | -0.35438 | 1.3853 | G2 |
| FAM107B | 4.39468 | 0.000529 | 9.18E-05 | 0.003151 | 1.026982 | -0.35303 | 1.380008 | G2 |
| IFIT2 | 5.18849 | 8.67E-05 | 2.82E-05 | 0.001683 | 1.023272 | -0.35175 | 1.375022 | G2 |
| APLP1 | 4.526223 | 0.000491 | 3.63E-05 | 0.001871 | 1.022221 | -0.35139 | 1.37361 | G2 |
| RTN1 | 5.040157 | 2.55E-05 | 0.000509 | 0.00897 | 1.021435 | -0.35112 | 1.372553 | G2 |
| LIMCH1 | 7.946433 | 2.18E-08 | 3.23E-06 | 0.000557 | 1.02015 | -0.35068 | 1.370827 | G2 |
| S100A1 | 6.094541 | 2.08E-06 | 5.56E-05 | 0.00239 | 1.017818 | -0.34988 | 1.367693 | G2 |
| MAP1A | 6.417578 | 1.83E-06 | 1.65E-05 | 0.00127 | 1.015582 | -0.34911 | 1.364689 | G2 |
| FBXL16 | 4.649594 | 0.000326 | 4.80E-05 | 0.002193 | 1.013841 | -0.34851 | 1.362349 | G2 |
| OTOS | 3.517634 | 0.005147 | 1.98E-05 | 0.001416 | 1.013401 | -0.34836 | 1.361757 | G2 |
| AMOT | 5.817138 | 1.41E-05 | 2.03E-05 | 0.001439 | 1.011501 | -0.3477 | 1.359204 | G2 |
| 8-Sep | 5.529728 | 0.000108 | 1.73E-06 | 0.000405 | 1.006905 | -0.34612 | 1.353029 | G2 |
| HOXD1 | 4.311323 | 0.000612 | 0.00012 | 0.003735 | 1.006076 | -0.34584 | 1.351915 | G2 |
| PRR18 | 4.256549 | 0.001461 | 2.89E-06 | 0.000538 | 1.00522 | -0.34554 | 1.350764 | G2 |
| SYN1 | 3.442703 | 0.005039 | 0.000118 | 0.003689 | 1.004389 | -0.34526 | 1.349648 | G2 |
| FA2H | 4.529342 | 0.000527 | 2.72E-05 | 0.001646 | 1.003089 | -0.34481 | 1.3479 | G2 |
| PPM1H | 6.988756 | 6.27E-06 | 3.85E-07 | 0.000317 | 0.99887 | -0.34336 | 1.342232 | G2 |
| SNAP25 | 3.08232 | 0.008957 | 0.000774 | 0.011834 | 0.995169 | -0.34209 | 1.337258 | G2 |
| RGS4 | 3.629276 | 0.002246 | 0.00092 | 0.013133 | 0.994566 | -0.34188 | 1.336448 | G2 |
| PRODH | 4.64562 | 8.17E-05 | 0.00091 | 0.013067 | 0.993593 | -0.34155 | 1.33514 | G2 |
| GNAO1 | 5.853053 | 2.09E-05 | 9.27E-06 | 0.000929 | 0.992897 | -0.34131 | 1.334205 | G2 |
| COLGALT2 | 4.443411 | 0.000206 | 0.000656 | 0.010679 | 0.992492 | -0.34117 | 1.333661 | G2 |
| CAMK2G | 7.235336 | 4.81E-06 | 2.27E-07 | 0.000317 | 0.983665 | -0.33813 | 1.3218 | G2 |
| PAMR1 | 4.185663 | 0.000757 | 0.000186 | 0.004781 | 0.976719 | -0.33575 | 1.312466 | G2 |
| LY86 | 6.159495 | 3.38E-06 | 2.52E-05 | 0.001589 | 0.974882 | -0.33512 | 1.309997 | G2 |
| IL1RAPL1 | 4.966071 | 5.95E-05 | 0.00022 | 0.005358 | 0.968895 | -0.33306 | 1.301953 | G2 |
| EPHB6 | 5.957113 | 4.40E-05 | 1.25E-06 | 0.000377 | 0.964466 | -0.33154 | 1.296001 | G2 |
| ASPA | 5.87564 | 1.14E-05 | 2.05E-05 | 0.001446 | 0.964017 | -0.33138 | 1.295398 | G2 |
| MYOM1 | 4.78177 | 0.000265 | 3.06E-05 | 0.001725 | 0.962433 | -0.33084 | 1.293269 | G2 |
| CES4A | 4.425938 | 0.000891 | 7.39E-06 | 0.000827 | 0.95833 | -0.32943 | 1.287755 | G2 |
| CX3CR1 | 3.666418 | 0.002013 | 0.000948 | 0.01342 | 0.954313 | -0.32805 | 1.282358 | G2 |
| EDIL3 | 4.789028 | 0.000542 | 1.75E-06 | 0.000405 | 0.953716 | -0.32784 | 1.281557 | G2 |
| KCNMB4 | 3.932497 | 0.001675 | 0.000104 | 0.003391 | 0.952173 | -0.32731 | 1.279483 | G2 |
| GSTO2 | 7.041137 | 4.44E-06 | 5.19E-07 | 0.000319 | 0.951859 | -0.3272 | 1.27906 | G2 |
| CBX7 | 6.643203 | 1.50E-06 | 8.31E-06 | 0.000868 | 0.945892 | -0.32515 | 1.271042 | G2 |
| SLC9A9 | 8.578193 | 2.28E-09 | 2.14E-06 | 0.000469 | 0.944258 | -0.32459 | 1.268847 | G2 |
| EVI2A | 5.572074 | 7.41E-05 | 3.57E-06 | 0.000582 | 0.938308 | -0.32254 | 1.260851 | G2 |
| PANX2 | 3.308415 | 0.006291 | 0.000217 | 0.005309 | 0.936382 | -0.32188 | 1.258264 | G2 |
| SYP | 3.554769 | 0.003535 | 0.000248 | 0.005737 | 0.935594 | -0.32161 | 1.257204 | G2 |
| FOLR2 | 4.425468 | 0.000176 | 0.000997 | 0.013884 | 0.933078 | -0.32075 | 1.253823 | G2 |
| SLC48A1 | 8.425083 | 5.64E-08 | 4.99E-07 | 0.000319 | 0.932556 | -0.32057 | 1.253123 | G2 |
| SPINT2 | 6.234887 | 3.60E-06 | 1.71E-05 | 0.001291 | 0.930347 | -0.31981 | 1.250154 | G2 |
| BOK | 4.332925 | 0.000934 | 1.71E-05 | 0.001293 | 0.924919 | -0.31794 | 1.24286 | G2 |
| LOC100129129 | 4.410878 | 0.000208 | 0.000802 | 0.012122 | 0.923314 | -0.31739 | 1.240703 | G2 |
| SLC8A2 | 3.436003 | 0.005005 | 0.000141 | 0.004047 | 0.921876 | -0.3169 | 1.238772 | G2 |
| ADARB2 | 4.370505 | 0.001176 | 2.70E-06 | 0.000529 | 0.920534 | -0.31643 | 1.236967 | G2 |
| RAB11FIP4 | 5.698286 | 4.37E-05 | 5.50E-06 | 0.000721 | 0.915298 | -0.31463 | 1.229932 | G2 |
| ROGDI | 5.618912 | 0.000138 | 4.19E-07 | 0.000319 | 0.914295 | -0.31429 | 1.228584 | G2 |
| STXBP6 | 4.160221 | 0.001276 | 2.63E-05 | 0.00161 | 0.912895 | -0.31381 | 1.226702 | G2 |
| ALDH2 | 7.358145 | 1.28E-08 | 2.94E-05 | 0.001704 | 0.912042 | -0.31351 | 1.225557 | G2 |
| SYNDIG1 | 5.125882 | 2.06E-05 | 0.000422 | 0.007999 | 0.911641 | -0.31338 | 1.225017 | G2 |
| OPTN | 7.530831 | 4.36E-08 | 7.06E-06 | 0.000808 | 0.910341 | -0.31293 | 1.22327 | G2 |
| RGS20 | 4.374775 | 0.000412 | 0.000226 | 0.005467 | 0.909257 | -0.31256 | 1.221815 | G2 |
| GFRA2 | 4.279665 | 0.000308 | 0.000912 | 0.013086 | 0.907786 | -0.31205 | 1.219837 | G2 |
| PLEKHG3 | 4.5117 | 0.000454 | 5.40E-05 | 0.002366 | 0.907072 | -0.31181 | 1.218877 | G2 |
| TLR7 | 4.821811 | 0.000253 | 2.55E-05 | 0.001595 | 0.90598 | -0.31143 | 1.217411 | G2 |
| TMC6 | 6.89328 | 1.59E-06 | 3.08E-06 | 0.000545 | 0.900197 | -0.30944 | 1.20964 | G2 |
| ABLIM1 | 5.263469 | 5.27E-05 | 4.73E-05 | 0.002177 | 0.898208 | -0.30876 | 1.206967 | G2 |
| TMEM130 | 3.51464 | 0.00416 | 0.000154 | 0.004249 | 0.892801 | -0.3069 | 1.199702 | G2 |
| SOWAHA | 3.786908 | 0.002441 | 8.66E-05 | 0.003043 | 0.892539 | -0.30681 | 1.199349 | G2 |
| RHOU | 4.978839 | 9.09E-05 | 9.65E-05 | 0.00328 | 0.891451 | -0.30644 | 1.197887 | G2 |
| CHN2 | 5.186113 | 0.000182 | 4.17E-06 | 0.000639 | 0.887583 | -0.30511 | 1.19269 | G2 |
| NIPA1 | 5.013683 | 0.000245 | 6.13E-06 | 0.000742 | 0.887156 | -0.30496 | 1.192115 | G2 |
| GSTM3 | 4.169762 | 0.000931 | 0.000102 | 0.003369 | 0.885382 | -0.30435 | 1.189732 | G2 |
| TP53INP2 | 3.865051 | 0.001732 | 0.000199 | 0.004993 | 0.882395 | -0.30332 | 1.185719 | G2 |
| NAPB | 3.445272 | 0.004997 | 0.00012 | 0.003745 | 0.87442 | -0.30058 | 1.175001 | G2 |
| SIK3 | 5.138212 | 9.30E-05 | 3.35E-05 | 0.001788 | 0.871522 | -0.29959 | 1.171107 | G2 |
| CRYAB | 4.9005 | 4.22E-05 | 0.000547 | 0.009433 | 0.871044 | -0.29942 | 1.170465 | G2 |
| KIAA0513 | 5.415122 | 7.27E-05 | 9.98E-06 | 0.000967 | 0.869977 | -0.29905 | 1.169032 | G2 |
| ASTN2 | 3.893448 | 0.001576 | 0.000219 | 0.005342 | 0.869847 | -0.29901 | 1.168857 | G2 |
| MYOT | 4.854633 | 0.000111 | 0.000145 | 0.004097 | 0.866698 | -0.29793 | 1.164626 | G2 |
| SLC6A1 | 4.953933 | 3.14E-05 | 0.000618 | 0.010265 | 0.865726 | -0.29759 | 1.163319 | G2 |
| TJP2 | 6.293976 | 2.15E-06 | 2.32E-05 | 0.001546 | 0.865474 | -0.29751 | 1.162981 | G2 |
| PKP4 | 4.385013 | 0.000478 | 0.000137 | 0.003965 | 0.862253 | -0.2964 | 1.158652 | G2 |
| NEBL | 6.631056 | 4.86E-06 | 2.28E-06 | 0.000488 | 0.862228 | -0.29639 | 1.158619 | G2 |
| ITPK1 | 5.995906 | 6.91E-06 | 2.26E-05 | 0.00153 | 0.859959 | -0.29561 | 1.15557 | G2 |
| RASL10A | 4.785911 | 0.000167 | 9.71E-05 | 0.003282 | 0.856816 | -0.29453 | 1.151347 | G2 |
| FEZ1 | 7.66993 | 8.75E-09 | 1.31E-05 | 0.001145 | 0.850867 | -0.29249 | 1.143352 | G2 |
| FTH1 | 5.331545 | 3.88E-05 | 5.44E-05 | 0.002369 | 0.849452 | -0.292 | 1.141451 | G2 |
| ARHGAP22 | 5.771165 | 3.83E-06 | 0.000125 | 0.003822 | 0.849049 | -0.29186 | 1.140909 | G2 |
| ACBD7 | 3.411587 | 0.004376 | 0.000542 | 0.009354 | 0.847042 | -0.29117 | 1.138212 | G2 |
| SLAIN1 | 3.991633 | 0.000792 | 0.000894 | 0.012955 | 0.845423 | -0.29061 | 1.136038 | G2 |
| TEX2 | 8.867422 | 3.77E-09 | 8.30E-07 | 0.000352 | 0.84528 | -0.29057 | 1.135845 | G2 |
| HS3ST2 | 3.675925 | 0.003286 | 6.18E-05 | 0.002529 | 0.843448 | -0.28994 | 1.133383 | G2 |
| SELM | 4.224062 | 0.000388 | 0.000831 | 0.012369 | 0.842458 | -0.28959 | 1.132053 | G2 |
| SEMA4D | 6.217094 | 1.34E-05 | 3.19E-06 | 0.000554 | 0.841873 | -0.28939 | 1.131268 | G2 |
| ZNF536 | 3.319244 | 0.006165 | 0.000211 | 0.005195 | 0.839965 | -0.28874 | 1.128703 | G2 |
| WFS1 | 6.095123 | 3.89E-06 | 2.86E-05 | 0.00169 | 0.838959 | -0.28839 | 1.127352 | G2 |
| SCD | 5.576662 | 1.18E-05 | 8.41E-05 | 0.002992 | 0.838352 | -0.28818 | 1.126535 | G2 |
| KCNJ10 | 5.853815 | 9.26E-06 | 3.01E-05 | 0.001713 | 0.838109 | -0.2881 | 1.126209 | G2 |
| TTYH2 | 6.435193 | 1.80E-05 | 6.93E-07 | 0.000336 | 0.837949 | -0.28805 | 1.125994 | G2 |
| CTNND2 | 5.404109 | 5.66E-06 | 0.000496 | 0.008811 | 0.837865 | -0.28802 | 1.125881 | G2 |
| PPP1R1A | 5.419496 | 5.18E-05 | 1.96E-05 | 0.00141 | 0.836663 | -0.2876 | 1.124267 | G2 |
| SNCG | 5.058674 | 0.000423 | 3.78E-07 | 0.000317 | 0.833508 | -0.28652 | 1.120026 | G2 |
| KNDC1 | 5.07804 | 0.000231 | 4.42E-06 | 0.000656 | 0.833418 | -0.28649 | 1.119905 | G2 |
| PIK3IP1 | 4.854299 | 0.000128 | 0.000108 | 0.003477 | 0.832388 | -0.28613 | 1.118521 | G2 |
| CECR1 | 5.458442 | 8.31E-06 | 0.000234 | 0.005546 | 0.831739 | -0.28591 | 1.117649 | G2 |
| GSN | 4.805994 | 8.47E-05 | 0.00032 | 0.006705 | 0.831058 | -0.28568 | 1.116734 | G2 |
| LILRA2 | 4.917644 | 0.000135 | 6.32E-05 | 0.002537 | 0.827377 | -0.28441 | 1.111788 | G2 |
| FAM171A1 | 7.938798 | 6.74E-09 | 6.70E-06 | 0.000784 | 0.825655 | -0.28382 | 1.109474 | G2 |
| ADD3 | 7.071033 | 1.60E-06 | 1.61E-06 | 0.000398 | 0.825653 | -0.28382 | 1.109472 | G2 |
| SLCO2B1 | 5.578051 | 4.34E-06 | 0.000276 | 0.006126 | 0.825201 | -0.28366 | 1.108864 | G2 |
| GABRG2 | 3.16268 | 0.008868 | 0.000154 | 0.004257 | 0.824014 | -0.28325 | 1.107269 | G2 |
| SYNM | 5.697487 | 1.47E-06 | 0.000563 | 0.009592 | 0.823082 | -0.28293 | 1.106016 | G2 |
| LYL1 | 7.47273 | 8.65E-08 | 5.27E-06 | 0.000721 | 0.822291 | -0.28266 | 1.104953 | G2 |
| NEFH | 3.348134 | 0.006002 | 0.000153 | 0.004241 | 0.820848 | -0.28217 | 1.103015 | G2 |
| FSTL5 | 5.201307 | 0.000285 | 6.72E-07 | 0.000334 | 0.816577 | -0.2807 | 1.097276 | G2 |
| SIGLEC8 | 4.133402 | 0.001055 | 8.97E-05 | 0.003114 | 0.815532 | -0.28034 | 1.095872 | G2 |
| MFSD6 | 4.744433 | 0.00015 | 0.000163 | 0.00438 | 0.812257 | -0.27921 | 1.09147 | G2 |
| ALDH4A1 | 7.221005 | 1.27E-06 | 1.22E-06 | 0.000377 | 0.811589 | -0.27898 | 1.090572 | G2 |
| DNAH17 | 4.217996 | 0.00158 | 2.84E-06 | 0.000535 | 0.809852 | -0.27839 | 1.088239 | G2 |
| WASF3 | 4.770514 | 8.76E-05 | 0.000376 | 0.007429 | 0.808904 | -0.27806 | 1.086965 | G2 |
| PLCXD3 | 6.794265 | 6.00E-06 | 8.84E-07 | 0.000353 | 0.808854 | -0.27804 | 1.086898 | G2 |
| P2RY12 | 3.407972 | 0.004807 | 0.000311 | 0.006592 | 0.808771 | -0.27802 | 1.086787 | G2 |
| SCN1B | 3.478163 | 0.005241 | 3.94E-05 | 0.001973 | 0.807093 | -0.27744 | 1.084531 | G2 |
| DYSF | 3.431346 | 0.004857 | 0.000195 | 0.004947 | 0.804933 | -0.2767 | 1.081628 | G2 |
| SH3BGRL2 | 6.288725 | 9.75E-07 | 5.15E-05 | 0.002302 | 0.801741 | -0.2756 | 1.07734 | G2 |
| DOCK10 | 5.085192 | 2.95E-05 | 0.000323 | 0.006741 | 0.801412 | -0.27549 | 1.076897 | G2 |
| LGI1 | 4.252581 | 0.000531 | 0.000306 | 0.006529 | 0.800456 | -0.27516 | 1.075613 | G2 |
| ZNF488 | 5.018813 | 4.58E-05 | 0.000245 | 0.005689 | 0.797265 | -0.27406 | 1.071324 | G2 |
| HAPLN2 | 3.994751 | 0.001698 | 4.39E-05 | 0.002089 | 0.79593 | -0.2736 | 1.06953 | G2 |
| CCDC85A | 6.519236 | 4.28E-05 | 6.45E-08 | 0.000317 | 0.795368 | -0.27341 | 1.068776 | G2 |
| C3 | 4.258271 | 0.000706 | 0.000121 | 0.003745 | 0.794121 | -0.27298 | 1.0671 | G2 |
| PRUNE2 | 4.492056 | 0.000318 | 0.000177 | 0.004671 | 0.793298 | -0.2727 | 1.065994 | G2 |
| ABCA8 | 4.985341 | 0.0001 | 7.65E-05 | 0.002842 | 0.792505 | -0.27242 | 1.064929 | G2 |
| EPHX1 | 7.464531 | 7.62E-09 | 2.96E-05 | 0.001705 | 0.789378 | -0.27135 | 1.060726 | G2 |
| SGIP1 | 5.542672 | 3.09E-05 | 2.48E-05 | 0.001589 | 0.788249 | -0.27096 | 1.05921 | G2 |
| KCNMA1 | 6.534387 | 7.01E-06 | 2.07E-06 | 0.000462 | 0.785522 | -0.27002 | 1.055545 | G2 |
| CADM3 | 4.415219 | 0.000268 | 0.000467 | 0.008557 | 0.784368 | -0.26963 | 1.053994 | G2 |
| SELL | 3.55281 | 0.004258 | 6.51E-05 | 0.002601 | 0.783911 | -0.26947 | 1.053381 | G2 |
| HPCA | 2.868923 | 0.014518 | 0.000633 | 0.010401 | 0.783623 | -0.26937 | 1.052993 | G2 |
| ACOT4 | 6.361087 | 9.13E-06 | 2.99E-06 | 0.000538 | 0.782086 | -0.26884 | 1.050928 | G2 |
| EMX2 | 4.501188 | 0.00033 | 0.000151 | 0.004203 | 0.78099 | -0.26847 | 1.049456 | G2 |
| NQO1 | 4.640246 | 0.000161 | 0.000284 | 0.006214 | 0.780068 | -0.26815 | 1.048216 | G2 |
| CA2 | 5.371598 | 8.41E-06 | 0.00036 | 0.007238 | 0.777638 | -0.26731 | 1.044951 | G2 |
| IFIT3 | 3.751767 | 0.001891 | 0.000487 | 0.008717 | 0.777201 | -0.26716 | 1.044363 | G2 |
| KLHL32 | 5.554841 | 6.43E-05 | 5.56E-06 | 0.000721 | 0.776132 | -0.2668 | 1.042927 | G2 |
| SLCO3A1 | 6.128197 | 1.91E-05 | 2.71E-06 | 0.000529 | 0.775453 | -0.26656 | 1.042015 | G2 |
| HERC6 | 5.218404 | 2.88E-05 | 0.000161 | 0.004345 | 0.775129 | -0.26645 | 1.04158 | G2 |
| SST | 3.692645 | 0.002851 | 0.000127 | 0.003834 | 0.774748 | -0.26632 | 1.041067 | G2 |
| ST18 | 3.975287 | 0.001205 | 0.000273 | 0.006084 | 0.774249 | -0.26615 | 1.040397 | G2 |
| LANCL1 | 5.312406 | 0.000118 | 6.00E-06 | 0.000736 | 0.772891 | -0.26568 | 1.038572 | G2 |
| MS4A7 | 3.967481 | 0.000911 | 0.000743 | 0.011554 | 0.763655 | -0.26251 | 1.026161 | G2 |
| LOC389033 | 3.63224 | 0.004413 | 8.55E-06 | 0.000879 | 0.760885 | -0.26155 | 1.02244 | G2 |
| LOC729680 | 5.146994 | 2.94E-05 | 0.000231 | 0.005502 | 0.758238 | -0.26064 | 1.018882 | G2 |
| CEBPA | 6.760645 | 1.05E-07 | 5.42E-05 | 0.002366 | 0.758233 | -0.26064 | 1.018876 | G2 |
| FGF1 | 5.967674 | 5.16E-05 | 8.09E-07 | 0.00035 | 0.757903 | -0.26053 | 1.018432 | G2 |
| CDC42EP2 | 5.168844 | 0.000321 | 5.27E-07 | 0.000319 | 0.757288 | -0.26032 | 1.017605 | G2 |
| ALAD | 5.83265 | 6.13E-05 | 1.16E-06 | 0.000377 | 0.753856 | -0.25914 | 1.012994 | G2 |
| KIAA0319 | 5.450047 | 4.07E-05 | 2.57E-05 | 0.001595 | 0.753639 | -0.25906 | 1.012702 | G2 |
| KIAA1045 | 4.146623 | 0.00155 | 9.61E-06 | 0.000945 | 0.753452 | -0.259 | 1.012452 | G2 |
| TUBA4A | 4.374369 | 0.000611 | 6.75E-05 | 0.002672 | 0.753216 | -0.25892 | 1.012135 | G2 |
| ENHO | 3.651461 | 0.002496 | 0.000453 | 0.008373 | 0.751806 | -0.25843 | 1.01024 | G2 |
| LPAR5 | 6.180325 | 4.73E-07 | 0.000165 | 0.004425 | 0.750998 | -0.25816 | 1.009153 | G2 |
| TMCC2 | 4.549621 | 0.000693 | 6.74E-06 | 0.000784 | 0.750588 | -0.25801 | 1.008603 | G2 |
| FOLH1 | 4.056366 | 0.001874 | 9.49E-06 | 0.000942 | 0.749792 | -0.25774 | 1.007533 | G2 |
| IRF8 | 5.136696 | 6.52E-05 | 6.82E-05 | 0.002692 | 0.747962 | -0.25711 | 1.005073 | G2 |
| FXYD1 | 4.444665 | 0.000323 | 0.000245 | 0.005689 | 0.746099 | -0.25647 | 1.002571 | G2 |
| SIRPA | 9.285478 | 1.39E-10 | 1.50E-06 | 0.000398 | 0.745908 | -0.25641 | 1.002314 | G2 |
| CDK18 | 3.841 | 0.001969 | 0.000142 | 0.004061 | 0.745798 | -0.25637 | 1.002166 | G2 |
| GPRC5B | 7.82775 | 4.71E-07 | 4.76E-07 | 0.000319 | 0.744795 | MGMT | 1.000818 | G2 |
| SHANK3 | 3.965559 | 0.001194 | 0.000311 | 0.006592 | 0.744365 | -0.25588 | 1.000241 | G2 |

*FDR: False Discovery Rate
