# Supplementary material for: Recurrent Glioblastomas Reveal Molecular Subtypes Associated with Mechanistic Implications of Drug-Resistance
Source: PLoS One. 2015 Oct 14;10(10):e0140528. doi: 10.1371/journal.pone.0140528 (PMC4605710; doi:10.1371/journal.pone.0140528)
Supplement: S2 Table — (DOC) [file pone.0140528.s007.doc]

### S2 Table. Classification of recurrent glioblastomas based on the gene expression changes from the paired primary tumors

| **Patient No.** | *** Hospital Code** | **Primary GBM** | **Original subtype**  **of primary tumor** | **Recurrent**  **GBM** | **Migrated subtype**  **by recurrence** | **Cluster Shift**  **upon recurrence** |
| --- | --- | --- | --- | --- | --- | --- |
| **G1R (G1 type recurrent glioblastomas without subtype migration)** | | | | | | |
| 1 | AJOU | GBM007 | G1 | GBM029 | G1 | G1 🡪 G1 |
| 2 | CNU | GBM036 | G1 | GBM037 | G1 | G1 🡪 G1 |
| 3 | CNU | GBM038 | G1 | GBM039 | G1 | G1 🡪 G1 |
| 4 | SNU | GBM042 | G1 | GBM048 | G1 | G1 🡪 G1 |
| 5 | SNU | GBM043 | G1 | GBM049 | G1 | G1 🡪 G1 |
| 6 | SNU | GBM046 | G1 | GBM052 | G1 | G1 🡪 G1 |
| **G2R (G2 type recurrent glioblastomas with subtype migration)** | | | | | | |
| 7 | AJOU | GBM003 | G1 | GBM009 | G2 | G1 🡪 G2 |
| 8 | AJOU | GBM006 | G1 | GBM012 | G2 | G1 🡪 G2 |
| 9 | CNU | GBM023 | G1 | GBM028 | G2 | G1 🡪 G2 |
| 10 | AJOU | GBM030 | G1 | GBM031 | G2 | G1 🡪 G2 |
| 11 | CNU | GBM034 | G1 | GBM035 | G2 | G1 🡪 G2 |
| 12 | SNU | GBM044 | G1 | GBM050 | G2 | G1 🡪 G2 |
| 13 | SNU | GBM045 | G1 | GBM051 | G2 | G1 🡪 G2 |
| **Unclassified** | | | | | | |
| 14 | CNU | GBM022 | G2 | GBM027 | G2 | G2 🡪 G2 |
| 15 | AJOU | GBM004 | G2 | GBM010 | G1 | G2 🡪 G1 |

* AJOU: Ajou University Hospital, CNU: Chonnam National University Hospital, SNU: Seoul National University Hospital
