# Supplementary material for: Recurrent Glioblastomas Reveal Molecular Subtypes Associated with Mechanistic Implications of Drug-Resistance
Source: PLoS One. 2015 Oct 14;10(10):e0140528. doi: 10.1371/journal.pone.0140528 (PMC4605710; doi:10.1371/journal.pone.0140528)
Supplement: S3 Table — (DOC) [file pone.0140528.s008.doc]

### S3 Table. Prediction of subtypes of glioblastomas by NTP

| **ID** | **prediction**  **Label*** | **Nominal P-value** | **FDR (BH)** | **Bonferroni p-value** |
| --- | --- | --- | --- | --- |
| GBM003 | 1 | 0.0002 | 0.000409 | 0.008598 |
| GBM006 | 3 | 0.0002 | 0.000409 | 0.008598 |
| GBM023 | 1 | 0.0002 | 0.000409 | 0.008598 |
| GBM030 | 4 | 0.0002 | 0.000409 | 0.008598 |
| GBM034 | 4 | 0.0008 | 0.000819 | 0.034393 |
| GBM044 | 3 | 0.0002 | 0.000409 | 0.008598 |
| GBM045 | 3 | 0.0002 | 0.000409 | 0.008598 |
| GBM004 | 2 | 0.0002 | 0.000409 | 0.008598 |
| GBM007 | 1 | 0.0002 | 0.000409 | 0.008598 |
| GBM022 | 2 | 0.0002 | 0.000409 | 0.008598 |
| GBM036 | 1 | 0.0002 | 0.000409 | 0.008598 |
| GBM038 | 5 | 0.0002 | 0.000409 | 0.008598 |
| GBM042 | 4 | 0.0002 | 0.000409 | 0.008598 |
| GBM043 | 4 | 0.0002 | 0.000409 | 0.008598 |
| GBM046 | 1 | 0.0002 | 0.000409 | 0.008598 |
| GBM001 | 2 | 0.0002 | 0.000409 | 0.008598 |
| GBM005 | 4 | 0.0002 | 0.000409 | 0.008598 |
| GBM008 | 1 | 0.0002 | 0.000409 | 0.008598 |
| GBM013 | 4 | 0.0002 | 0.000409 | 0.008598 |
| GBM014 | 3 | 0.0002 | 0.000409 | 0.008598 |
| GBM015 | 4 | 0.0002 | 0.000409 | 0.008598 |
| GBM016 | 4 | 0.0002 | 0.000409 | 0.008598 |
| GBM017 | 3 | 0.0002 | 0.000409 | 0.008598 |
| GBM018 | 1 | 0.0002 | 0.000409 | 0.008598 |
| GBM021 | 3 | 0.0002 | 0.000409 | 0.008598 |
| GBM025 | 1 | 0.0002 | 0.000409 | 0.008598 |
| GBM009 | 2 | 0.0002 | 0.000409 | 0.008598 |
| GBM012 | 2 | 0.0002 | 0.000409 | 0.008598 |
| GBM028 | 2 | 0.0002 | 0.000409 | 0.008598 |
| GBM031 | 4 | 0.0002 | 0.000409 | 0.008598 |
| GBM035 | 3 | 0.017197 | 0.017197 | 0.739452 |
| GBM050 | 2 | 0.0002 | 0.000409 | 0.008598 |
| GBM051 | 2 | 0.0002 | 0.000409 | 0.008598 |
| GBM010 | 1 | 0.0002 | 0.000409 | 0.008598 |
| GBM029 | 1 | 0.0002 | 0.000409 | 0.008598 |
| GBM027 | 4 | 0.0002 | 0.000409 | 0.008598 |
| GBM037 | 4 | 0.0002 | 0.000409 | 0.008598 |
| GBM039 | 3 | 0.0002 | 0.000409 | 0.008598 |
| GBM048 | 4 | 0.0002 | 0.000409 | 0.008598 |
| GBM049 | 1 | 0.0002 | 0.000409 | 0.008598 |
| GBM052 | 1 | 0.0002 | 0.000409 | 0.008598 |
| GBM011 | 4 | 0.0002 | 0.000409 | 0.008598 |
| GBM026 | 1 | 0.0002 | 0.000409 | 0.008598 |

* Numeric code for 1, 2, 3, and 4 are indicated for proneural, neural, classical, and mesenchymal makers, respectively, and 5 for the REST.
