# Supplementary material for: Recurrent Glioblastomas Reveal Molecular Subtypes Associated with Mechanistic Implications of Drug-Resistance
Source: PLoS One. 2015 Oct 14;10(10):e0140528. doi: 10.1371/journal.pone.0140528 (PMC4605710; doi:10.1371/journal.pone.0140528)
Supplement: S4 Table — (DOC) [file pone.0140528.s009.doc]

### S4 Table. Mutations of drug resistance-related genes in TCGA data

| **Gene** | ***MAF Files** | **Mutation Frequency** | |
| --- | --- | --- | --- |
|  |  | **G1-like subtype (n=25)** | **G2-like subtype (n=23)** |
| TP53 | UCSC IlluminaGA DNASeq automated | 4 | 4 |
|  | BI IlluminaGA DNASeq | 5 | 5 |
| MGMT | UCSC IlluminaGA DNASeq automated | 0 | 0 |
| MSH6 | BI IlluminaGA DNASeq | 0 | 1 |
| ABCC1 | UCSC IlluminaGA DNASeq automated | 1 | 0 |
|  | BI IlluminaGA DNASeq | 1 | 0 |
| ABCC5 | UCSC IlluminaGA DNASeq automated | 0 | 0 |
|  | BI IlluminaGA DNASeq | 0 | 0 |

*MAF : Mutation Annotation Format

Somatic mutation data was downloaded from TCGA data portal (<https://tcga-data.nci.nih.gov/tcga/tcgaHome2.jsp>). To calculate mutation frequency in each gene, two types of mutation information MAF file, UCSC IlluminaGA DNASeq automated and BI lluminaGA DNASeq, were used with following criteria.; In case of UCSC IlluminaGA DNASeq automated MAF file, we only count the mutation event which is not classified as “silent” in the variant classification column and as “novel” in the “Dbsnp_Rs” column. And in case of BI lluminaGA DNASeq MAF file, we only consider mutation event which was not classified as “silent” in the “variant classification” column. Tumors with at least one mutation event were counted in each subtype.
